# Genomic Identification and Functional Characterization of Essential Genes in *Caenorhabditis elegans*

**DOI:** 10.1534/g3.117.300338

**Published:** 2018-01-15

**Authors:** Zhaozhao Qin, Robert Johnsen, Shicheng Yu, Jeffrey Shih-Chieh Chu, David L. Baillie, Nansheng Chen

**Affiliations:** * Department of Molecular Biology and Biochemistry, Simon Fraser University, Burnaby, British Columbia V5A 1S6, Canada; † Key Laboratory of Combinatorial Biosynthesis and Drug Discovery, School of Pharmaceutical Sciences, Wuhan University, 430071, China; ‡ Wuhan Frasergen Bioinformatics, Wuhan East Lake High-Tech Zone, 430075, China

**Keywords:** essential gene, lethal, genetic balancer, whole genome sequencing (WGS), functional characterization

## Abstract

Using combined genetic mapping, Illumina sequencing, bioinformatics analyses, and experimental validation, we identified 60 essential genes from 104 lethal mutations in two genomic regions of *Caenorhabditis elegans* totaling ∼14 Mb on chromosome III(mid) and chromosome V(left). Five of the 60 genes had not previously been shown to have lethal phenotypes by RNA interference depletion. By analyzing the regions around the lethal missense mutations, we identified four putative new protein functional domains. Furthermore, functional characterization of the identified essential genes shows that most are enzymes, including helicases, tRNA synthetases, and kinases in addition to ribosomal proteins. Gene Ontology analysis indicated that essential genes often encode for enzymes that conduct nucleic acid binding activities during fundamental processes, such as intracellular DNA replication, transcription, and translation. Analysis of essential gene shows that they have fewer paralogs, encode proteins that are in protein interaction hubs, and are highly expressed relative to nonessential genes. All these essential gene traits in *C. elegans* are consistent with those of human disease genes. Most human orthologs (90%) of the essential genes in this study are related to human diseases. Therefore, functional characterization of essential genes underlines their importance as proxies for understanding the biological functions of human disease genes.

Genes that are required for survival, or genes that strongly contribute to fitness and robust competitive growth are essential genes (Gerdes *et al.* 2006). In humans, it has been demonstrated that mutations in essential genes contribute to a spectrum of human diseases, from developmental diseases (often resulting in spontaneous abortions) to a range of cancers, including brain and breast cancers (Dickerson *et al.* 2011). Knowing what essential genes are and how they work will allow researchers to develop a deeper understanding of the molecular nature of such diseases. Identification of essential genes should also help to determine the minimal gene set, which is the smallest group of genes that are necessary and sufficient to sustain a functioning cell or organism under the most favorable conditions [reviewed in Koonin (2000)]. Thus, identifying essential genes will further our understanding of the basics of cell functioning.

Studies of essential genes are ubiquitous in model organisms. In mouse models using individual knockout strains, ∼2000 genes have been classified as essential. The deletion of these genes leads to either lethality before reproduction, or to sterility (Liao and Zhang 2007). In zebrafish, ∼390 genes have been identified through mutagenesis screens as essential for embryonic and early larval development (Amsterdam *et al.* 2004). In *C. elegans*, genome-wide functional analysis, using RNAi, indicates that at least 1170 genes are essential (Kamath *et al.* 2003). Although RNAi is an effective high-throughput technology for identifying essential genes, this technology has limitations that prevent it from being used to identify all essential genes in *C. elegans* (Kamath *et al.* 2003).

Genome-wide forward genetic screens are capable of isolating genes that play important roles in given phenotypes, including lethality. For over 35 yr, researchers have taken advantage of genetic balancers and forward genetic screens to isolate lethal mutations and identify essential genes in *C. elegans*. Using traditional genetic methods, including genetic mapping and transgenic rescue assays, many essential genes have been identified. However, due to the limitations of traditional genetic methods, the process of identifying essential genes is slow.

Here we used whole genome sequencing (WGS) technology to speed up the identification of essential genes in *C. elegans* mutants isolated from genetic screens (Rosenbluth and Baillie 1981; Rosenbluth *et al.* 1983, 1985; Johnsen and Baillie 1991; Stewart *et al.* 1998). Janke *et al.* (1997) was a harbinger of our work when they used sequenced cosmids to rescue lethal phenotypes on chromosome III and thus molecularly identify essential genes. In *C. elegans*, WGS is capable of capturing molecular lesions that were induced by ethyl methane sulfonate (EMS) mutagenesis in both homozygous (Sarin *et al.* 2008; Rose *et al.* 2010) and heterozygous backgrounds (Chu *et al.* 2012, 2014). Along with bioinformatics analysis, different types of mutations can be detected, such as base pair alterations, direct repeat sequences, and deletions, as well as insertions of transposable elements (Rose *et al.* 2010). However, to decide which mutation is responsible for a lethal phenotype, genetic mapping evidence, used for assigning sequenced lethal mutations into individual genetic mapping zones, proved to be indispensable. The mapping zones were defined via complementation tests between sets of overlapping deficiencies and duplications. Once zones were established lethals were complementation tested against those rearrangements to determine which zone each lethal fell into. After that, the lethals were complementation tested against lethals in the same zone to determine if they were allelic or newly identified genes (Rosenbluth and Baillie 1981; Rosenbluth *et al.* 1988; Johnsen and Baillie 1991; Stewart *et al.* 1998). Since the breakpoints of several deficiencies on both chromosome III(mid) and chromosome V(left) have been precisely located in a physical map, the boundaries of the corresponding genetic mapping zones are also well defined (Jones *et al.* 2007, 2009) thus narrowing considerably the molecular regions where the genes must reside. Methods such as single nucleotide polymorphism mapping (Davis *et al.* 2005) or a recently devised molecular inversion probes–based method (Mok *et al.* 2017) are necessary when the approximate position of the mutations are not known. In our case, with well-mapped mutations, the bioinformatics analysis needed to discover the exact positions of the recovered lethal lesions is simpler.

Homozygous mutations have a 100% allelic ratio, but lethal mutations need to be maintained as heterozygotes, which increases the difficulty of identifying lethal mutations. Furthermore, different balancer systems give rise to different heterozygous allelic ratios. For example, there are two copies of a recessive lethal mutation in a duplication-balanced region and one wild-type allele on the balancer. As a result, the allele frequency is two mutations to one wild type, which should result in a 66.7% variation frequency in the sequencing data. For translocations, only one copy of a lethal allele and one copy of a wild-type allele are present, and the variation frequency should be 50%. It has been demonstrated that duplication balanced essential genes can be identified when there is a 2:1 mutation-to-wild type allele ratio (Chu *et al.* 2012, 2014); however, when the ratio is 1:1 (using a translocation balancer), the identification becomes more difficult. In this study, we used genetic mapping data, WGS techniques, bioinformatics analyses, and experimental validation, to identify 60 essential genes from 104 lethal mutations in *C. elegans*. This was followed by functional characterization of those essential genes. We also studied the relationship between gene essentiality and gene duplicability, protein connectivity, gene expression, and showed that compared with nonessential genes, essential genes have fewer paralogs, encode proteins that are in interaction hubs, and are more highly expressed. The essential genes we identified provide a rich resource for future studies. The conserved essential genes should also prove useful for understanding the functions of homologous genes in human, especially disease-related genes. In addition, species-specific essential genes could be good candidates for targeting pathogenic nematodes.

## Materials and Methods

### C. elegans strains

All the strains that were submitted for sequencing and for complementation tests are included in the Supplemental Material, Table S2, Table S3, and Table S4 in File S1.

### WGS and computational analysis

Worms were grown on large (100 by 15 mm) Petri plates until the worms starved or the plates were covered in healthy gravid adults. We rinsed the worms off the plates at room temperature with M9 buffer (6 g Na_2_HPO_4_, 3 g KH_2_PO_4_, 5 g NaCl, 0.2 g MgSO_4_ in 1 liter of H_2_0) into 15 ml polypropylene tubes, and pelleted them by centrifuging at 1500 rpm for 2 min at 4°. We then washed them in 12 ml iced M9 buffer, for three times. The worms were placed on a rocker for 2–3 hr at room temperature to digest bacteria. Worms were pelleted at 1500 rpm for 2 min at 4°. Then, the supernatant was removed and the worms were washed and pelleted in 4 ml room temperature M9 buffer. The pellets were frozen at −80°.

The genomic DNA collection for Illumina sequencing was constructed using the QIAGEN DNeasy Blood & Tissue Kit (cat. no. 69504), following the protocol “Purification of Total DNA from Animal Tissues (Spin-Column Protocol).” DNA concentrations were determined using the Qubit dsDNA High Sensitivity Assay Kit performed with the Qubit 2.0 Fluorometer (Life Technologies). The purity of the DNA was assessed using the Nanodrop ND-1000 Spectrophotometer-T* (Thermo Scientific). For each sample, 0.6–2 μg of purified genomic DNA was submitted for sequencing at the British Columbia Cancer Agency Canada’s Michael Smith Genome Sciences Centre using the Illumina PET HiSeq technology.

### WGS data analysis

For chromosome III, genomic DNA libraries of 49 strains were prepared and sequenced using Illumina PET HiSeq to generate 100-bp-long paired end reads. BWA (Li and Durbin 2009), GATK (McKenna *et al.* 2010), and SAMtools (Li *et al.* 2009) were used to align the reads to the *C. elegans* reference genome (WS246) and called for variants. For chromosome V, the sequencing reads were first aligned with the *C. elegans* reference genome using BWA with default settings. WormBase WS249 was used as reference genome for the alignment. This step was followed by filtering out PCR-caused duplicates by using SAMtools (Li *et al.* 2009), which was also used for analyzing the sequencing depth of each strain. The breakpoints of large deletions, medium insertions, and translocations were detected and viewed using the Integrative Genomics Viewer (Robinson *et al.* 2011; Thorvaldsdottir *et al.* 2013). The effect of the variations on each CDS in the genome was analyzed using CooVar (Vergara *et al.* 2012).

### PCR sequencing and the rescue assay

Primers were designed using Primer3 (Koressaar and Remm 2007; Untergasser *et al.* 2012) to amplify the coding sequences of each candidate essential gene. PCR products were subjected to PCR purification (QIAGEN MinElute PCR Purification Kit, cat. no. 28004) or gel purification (QIAGEN QIAquick Gel Extraction Kit, cat. no. 28704) before they were sequenced using the Sanger technique. The same primers that were used in PCR amplification were also used for Sanger sequencing. The sequencing results were analyzed using the software SeqMan (DNASTAR).

Fosmid DNA was prepared using the Pharmacia Mini-Prep Kit Plus (minus the final column purification step) and was diluted in ddH_2_0. Primers were designed and PCR amplifications were performed to validate the presence of the candidate genes in the tested fosmids and their absence in the negative control fosmids.

The transgenic strains constructed for this research used the coinjection semidominant marker *rol-6*(*su1006*) (Kramer *et al.* 1990) in plasmid pCes1943 and another co-injected GFP marker *Pmyo-2*::*GFP*, which is expressed in the pharyngeal muscle (Dibb *et al.* 1989). DNA mixtures that contain 2 ng fosmid DNA, 80 ng *rol-6*(*su1006*), and 2.5 ng *Pmyo-2*::*GFP* were directly injected into the syncytial gonad of young adults of the mutant hermaphrodites (Mello *et al.* 1991). The presence of DpyUnc F_2_ progeny with coinjected markers indicated successful rescue.

### Interallelic complementation tests

Complementation tests were carried out for genetic validation of the candidate essential genes. For lethals balanced by *sDp3*, Unc-32 hermaphrodites were crossed to N2 males. Phenotypically wild-type F_1_ males were then crossed to another lethal with the same genetic background. The presence of fertile DpyUnc F_2_ hermaphrodites indicates complementation, while the absence of fertile DpyUnc F_2_ hermaphrodites indicates failure to complement (Stewart *et al.* 1998). For mutants balanced with *eT1*, phenotypically wild-type males containing a lethal mutation on chromosome V were crossed to another *eT1* balanced lethal. The presence of fertile DpyUnc F_1_ progeny indicates complementation, while the absence of fertile DpyUnc progeny indicates failure to complement. In some cases, late blocking, sterile, or maternal effect individual F_1_s were set up in order to observe the terminal phenotypes (Johnsen and Baillie 1991).

### Essential genes functional analyses

#### Protein domain analysis:

A known annotated domain for each protein was searched with InterProScan (Mulder and Apweiler 2007) using the Pfam database (Finn *et al.* 2014) and visualized with the Perl module FeatureStack (Frech *et al.* 2012). Identification of orthologs of *C. elegans* proteins was conducted using InParanoid (Remm *et al.* 2001) in 25 other nematode species and four model organisms: *Homo sapiens*, *Drosophila melanogaster*, *Mus musculus*, and *Danio rerio*. This was followed by multiple sequence alignments with ClustalX2 (Thompson *et al.* 1997; Larkin *et al.* 2007) between *C. elegans* proteins and their orthologs. The alignment was examined using Jalview version 2 with the “ClustalX” color scheme.

#### Gene Ontology analysis:

Gene Ontology (GO) was performed using the PANTHER classification system (Mi *et al.* 2013) from the website http://pantherdb.org/. Three GO term categories (cellular component, biological process, and molecular function) were examined individually.

#### Gene duplicability:

An all-against-all BLASTP search was conducted for the whole set of *C. elegans* proteins (WS250). Only the longest isoform was used if there are multiples transcripts of a gene. This was followed by the computation of the global PID. Only protein pairs that had a PID equal to or higher than 50% were kept and sorted by *e*-value.

#### Protein connectivity:

The whole genome protein interactions in *C. elegans* were downloaded from BioGRID (Stark *et al.* 2006), which is an interaction repository with data compiled through comprehensive curation efforts. BioGRID currently holds >8037 nonredundant interactions in *C. elegans* with 3949 unique genes that were derived from 205 publications, including both physical and genetic evidence. This was followed by the filtration of proteins that are from one of four groups.

#### Gene expression:

Gene expression data were downloaded through the *GExplore* (version 1.4) expression search interface (Hutter *et al.* 2009), which includes a series of *C. elegans* developmental stages from parts of the NHGRI modENCODE project (Hillier *et al.* 2009; Gerstein *et al.* 2010). The data were derived from synchronized whole animals from embryonic and postembryonic stages, followed by RNA-seq.

### Data availability

Strains are available upon request. Table S1 in File S1 contains detailed information of all identified essential genes and lethal mutations. Table S2 in File S1 includes a total of 86 strains with *eT1*(*III*;*V*) that were sequenced. Table S3 in File S1 contains a total of 49 strains with *sDp3* sequenced. Table S4 in File S1 includes all the strains used in the complementation tests. The WGS data of all 135 sequenced strains are publicly available on the NCBI with the BioProject accession number PRJNA416306.

## Results

### Balancer systems

**Figure 1:**
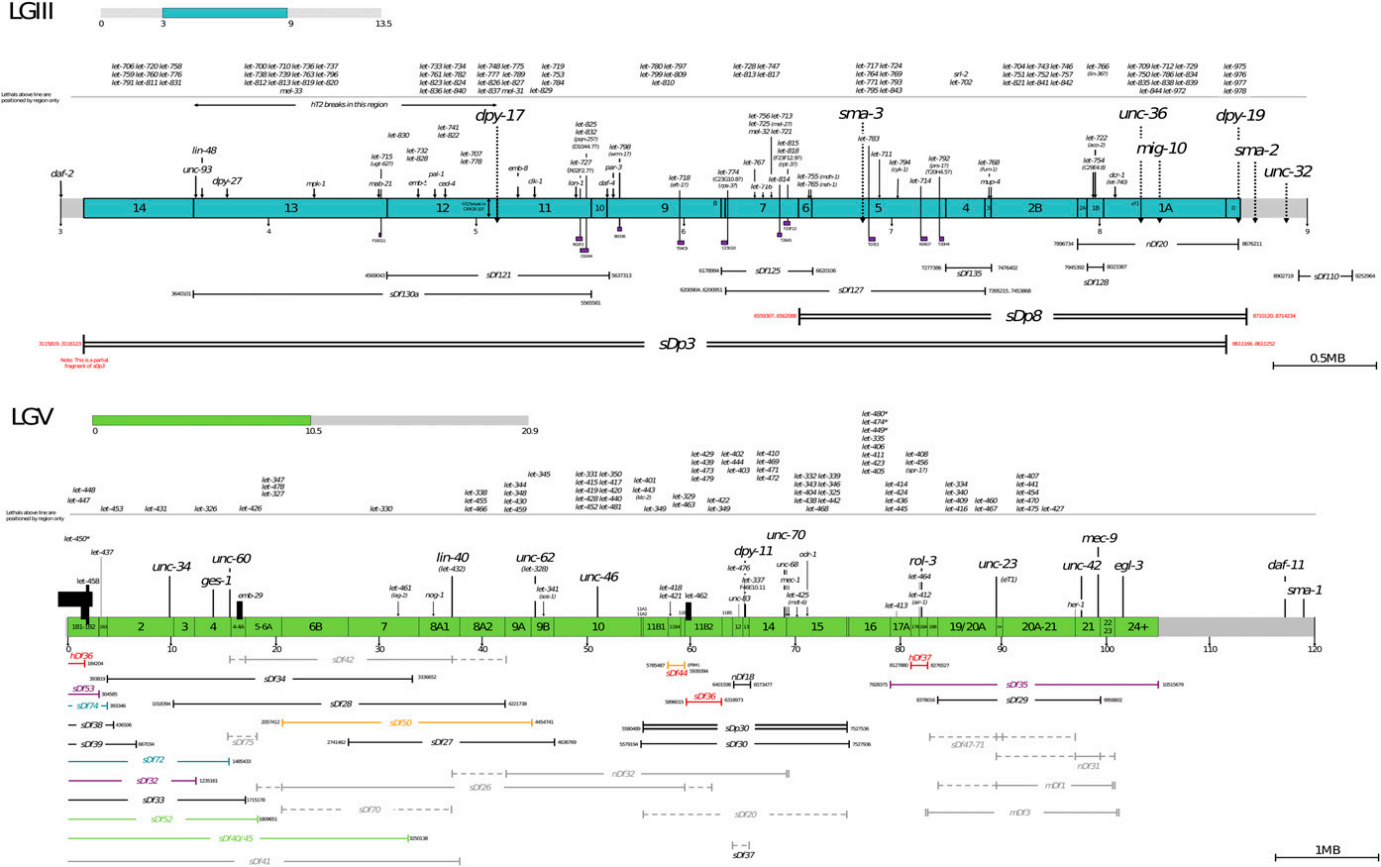
Physical deficiency map of *LGIII*(mid) and *LGV*(left). The top figure shows the middle region (4.5 Mb) of chromosome III that is balanced by a free duplication (*sDp3*), which has been subdivided into 14 main zones. The bottom figure shows the left half of chromosome V that is balanced by a reciprocal translocation (*eT1*), which has been subdivided into 22 main zones and several additional subzones by sets of overlapping rearrangements (deficiencies and one duplication).

Lethal mutations isolated from two balancers systems were used in our study: *dpy-18*(*e364*)*/eT1*(*III*); *unc-46*(*e177*)*/eT1*(*V*) and *dpy-17*(*e164*) *unc-32*(*e189*)*/dpy-17*(*e164*) *lin-12*(*n941*) *III*;*sDp3* (*III*;*f*). The first, the eT1-system, is a reciprocal translocation between the right half of chromosome III [*LGIII*(right)] and the left half of chromosome V [*LGV*(left)], recombinationally balancing those two regions (Rosenbluth and Baillie 1981), which is ∼20% of *C. elegans*’ genome. *LGV*(left) contains ∼7% (23 map units) of the recombination distance in the genome and ∼10% of its DNA. *LGV*(left) has been subdivided into 22 main zones and several additional subzones by sets of overlapping rearrangements (deficiencies and one duplication) (see Figure 1 courtesy of M. Jones) (Johnsen and Baillie 1988, 1991; Rosenbluth *et al.* 1988; Clark *et al.* 1990; Stewart *et al.* 1991). Over 120 nonessential (Edgley and Riddle 1990) and essential genes have been mapped to the zones in *LGV*(left), making it a genetically well-defined region. A number of different types of mutagens have been characterized in the eT1-system including formaldehyde (Johnsen and Baillie 1988), ultraviolet irradiation (UV) (Stewart et al. 1991), the transposable element Tc1 (Clark et al. 1990), and also EMS, which induces primarily point mutations (Rosenbluth *et al.* 1983; Johnsen and Baillie 1991), and other mutations that generally induce rearrangements γ-irradiation (Rosenbluth *et al.* 1985). *dpy-18*(*e364*)*/eT1*(*III*); *unc-46*(*e177*)*/eT1*(*V*) is phenotypically wild type; pseudolinked *dpy-18*; *unc-46* have a dumpy uncoordinated (Dpy-18 Unc-46) phenotype while *eT1*(*III*) breaks in *unc-36*, thus giving homozygous *eT1* a visible Unc-36 (uncoordinated) phenotype. Screening for the absence of fertile Dpy-18 Unc-46 indicates a lethal mutation in the balanced regions, after which the lethal mutations can be mapped to either *LGIII* or *LGV*. Those mutations on *LGV* can be complementation tested against the set of overlapping rearrangement to determine the zone in which the lethal mutation lies. Further complementation tests against genes in that zone determine which gene the mutation is allelic to.

The second system (Stewart *et al.* 1998) uses a free duplication (*sDp3*) (which does not recombine with the normal *LGIII* chromosome) to balance a 4.5 Mb region of *LGIII* (see Figure 1). The *sDp3* system consists of *dpy-17*(*e164*) *unc-32*(*e189*)*/dpy-17*(*e164*) *lin-12*(*n941*) *III*;*sDp3* (*III*;*f*) in which *sDp3* covers *dpy-17* (which has a dumpy phenotype) (*lin-12* is also covered by *sDp3* and has a visible phenotype) but not *unc-32* (which has an uncoordinated phenotype), so when the duplication is present over *dpy-17*(*e164*) *unc-32*(*e189*), the phenotype is Unc-32 but when absent it is Dpy-17 Unc-32. *dpy-17*(*e164*) *unc-32*(*e189*)*/dpy-17*(*e164*) *lin-12*(*n941*) *III*;*sDp3* (*III*;*f*) (which has a wild-type phenotype) was mutagenized and the F_2_ were screened for the absence of Dpy-17 Unc-32 progeny, indicating a putative lethal mutation in the *sDp3* balanced region on the *dpy-17*(*e164*) *unc-32*(*e189*) marked chromosome, *i.e.*, *dpy-17*(*e164*) *let-x unc-32*(*e189*)*/dpy-17*(*e164*) *let-x unc-32*(*e189*) *III*;*sDp3* (*III*;*f*). Strains were maintained as homozygotes with *sDp3* by picking Unc-32. The *sDp3* balanced region has also been divided into zones by a set of overlapping rearrangements. Stewart *et al.* (1998) reported 112 essential genes in the *sDp3* balanced region. A difference between the two systems is that in the eT1-system, the lethals were isolated as heterozygotes whereas in the sDp3-system the lethals were picked up as homozygotes covered by duplication. We did not note any obvious differences in the types of genes isolated by the two systems.

### Identification of genomic variations in chromosome III mutations balanced by sDp3

Genomic DNA libraries for 49 lethals (*dpy-17*(*e164*) *let-x unc-32*(*e189*);*sDp3* (*III*;*f*)) were prepared and sequenced using Illumina PET HiSeq to generate 100 bp paired end reads. We used BWA (Li and Durbin 2009), GATK (McKenna *et al.* 2010), and SAMtools (Li *et al.* 2009) to align the reads to a *C. elegans* reference genome (WS246) and called for variants. The average coverage for the 49 strains was 25×, with the lowest being 12× and the highest being 38×. The lethal mutations on chromosome III were balanced by *sDp3* and thus we expected the variant frequencies to be ∼67%. Single nucleotide variations (SNVs) that occurred with variant frequencies between 40 and 90% were selected from the 49 strains and subjected to three filtration steps. First, mutations found as part of the Million Mutation Project (MMP) are presumptively false positives, since the way MMP was designed should drastically reduce but not eliminate the chances of capturing lethal mutations (Flibotte *et al.* 2010; Thompson *et al.* 2013). One possible way the MMP could capture a lethal mutations is to capture a corresponding suppressor, but our initial assumption is “presumptive false positives.” Second, some variations (background variations) could stem from the starting strain that was used for mutagenesis. To be cost-effective, we did not sequence the starting strain because the majority of the *sDp3* balanced strains stemmed from it, thus they shared the same genetic background; instead, we assumed all variations that occurred multiple times in the 49 sequenced strained originated in the starting strain and excluded them. Third, we required the variations to be supported by at least eight reads in both forward and reverse directions. After the three steps of filtration the remaining SNVs were subjected to further lethal mutation identification Table 1.

**Table 1:** Average number of SNVs per strain with 20–100% variation frequency in the *eT1(V)* balanced region, before and after the removal of background variations

	Before Removal of Background Mutations/Strain	After Removal of Background Mutations/Strain
Chromosome V *eT1*	1357	72
Genetic coding region	282	13

### Identification of genomic variations in eT1 balanced chromosome V mutations

Before identifying the genomic variations in the *eT1* balanced mutations, there was a technical challenge that we needed to address. The progeny of these mutants include both phenotypically wild-type animals, which are *dpy-18*(*e364*)*/eT1*(*III*); *let-x unc-46*(*e177*)*/eT1*(*V*), and Unc-36 *eT1*(*III*;*V*) animals (because eT1 breaks in *unc-36*). These homozygous Unc-36 worms are fertile and contribute to the total sequenced genomic DNA, which makes the allelic ratios of lethal mutations very difficult to predict and effectively impossible to analyze (D. L. Baillie, unpublished data). An important step is to drastically minimize the contribution of *eT1* DNA so that the majority of sequenced DNA comes from *dpy-18*(*e364*)*/eT1*(*III*); *let-x unc-46*(*e177*)*/eT1*(*V*), allowing the allelic ratio of lethal mutations to approach 50% and thus facilitate the identification of lethal mutations. To do this, we constructed new strains for each mutant using *let-500*(*s2165*), which is located on the *eT1* balancer resulting in *dpy-18*(*e364*)*/eT1*(*III*); *unc-46*(*e177*) *let-x/let-500*(*s2165*) *eT1*(*V*). *let-500*(*s2165*) blocks development early and thus drastically reduces the contribution of homozygous *eT1* DNA to our sequence analyzes. This step proved extremely effective and useful for the identification of heterozygous mutations.

Genomic DNA libraries for 86 mutants were prepared and sequenced using Illumina PET HiSeq to generate 150 bp paired end reads. BWA (Li and Durbin 2009) and SAMtools (Li *et al.* 2009) were used to align the paired end reads to the WS249 *C. elegans* reference genome and postalignment processing analysis, respectively. This yielded an average of 31-fold genome coverage per sample (lowest coverage 23-fold, highest coverage 44-fold). We then used VarScan2 (Koboldt *et al.* 2012) to identify SNVs that occurred with variant frequencies between 20 and 100%, which was followed by three filtration steps as used in the identification of variations in the *sDp3* balanced region. After filtration, the numbers of remaining SNVs are summarized in Table 1.

The 86 sequenced strains have four different visible markers. Two (*dpy-18*(*e364*)*III* and *unc-46*(*e177*)*V*) were molecularly identified before and successfully validated in this study. Both those visible markers are heterozygous and balanced by *eT1*(*III*;*V*), and the sequencing data showed average variation frequencies of *e364* and *e177* are 50 and 51%, respectively. Our analysis also identified the previously unknown mutations in the other two *eT1*(*III*;*V*) balanced visible markers (*unc-42*(*e270*)*V* and *unc-60*(*e677*)*V*). We curated their mutation types and genomic positions: *e270* (A->T at position 976,514) causes a valine to glutamic acid change in four isoforms of *unc-42*; and *e677* (G->A at position 1,476,888) causes an amino acid change from glycine to arginine, which inhibits the normal function of the muscle-specific isoform UNC-60B.

### Identification of essential genes and experimental validation

Using the identified genomic variations, we performed essential gene identification and experimental validation for both chromosome III(mid) and chromosome V(left).

For the chromosome III(mid) dataset, 49 mutants with 49 lethal mutations representing 43 genes were sequenced. Six genes had two sequenced alleles while 37 genes had single alleles sequenced. The 49 mutations had previously been assigned to 14 genetic mapping zones (Table S3 in File S1). The 86 mutants from chromosome V(left) had previously been mapped to 23 zones (Table S2 in File S1), representing 46 genes. Two alleles were sequenced for 40 of the 46 genes while only six genes had a single allele sequenced. In total, there are 89 essential genes from chromosome III(mid) and chromosome V(left) that need to be identified molecularly.

The molecular identification of essential genes on chromosome III(mid) and chromosome V(left) was determined by using five lines of evidence. First, previous genetic mapping data narrowed down the location of the lethal mutation for each strain (Rosenbluth and Baillie 1981; Rosenbluth *et al.* 1988; Johnsen and Baillie 1991; Stewart *et al.* 1998). Second, bioinformatics analyses identified a limited number of variations in the genetically determined regions (Table 1). Third, there are two independent sequenced alleles for most of the genes analyzed, which not only makes genomic identification easier but the result for each allele provides validation for the other allele. Fourth, we used WormBase information about lethal phenotypes, including those supported by RNAi (www.wormbase.org), to further narrow our search for variations in our sequences. Last, the MMP dataset was used to help verify possible essential genes. In MMP, 2007 strains were subjected to mutagenesis and >800,000 different mutations were recovered in 20,115 genes. The large number of variations provides an unprecedented genetic resource for *C. elegans* research. However, during the MMP genetic screens only mutants with nonlethal phenotypes were isolated, and so most chain-terminating mutations such as the majority of nonsense mutations or splicing mutations in essential genes would be selected against (Thompson *et al.* 2013). In general, only missense mutations that do not cause malfunctioning proteins would be recovered. An essential gene example is the ribosomal large subunit *rpl-22*, for which MMP recovers only missense mutations. Using this hypothesis, we checked candidate genes by searching the MMP database for lack of chain-terminating mutations. With the above methodology we identified, with high confidence, 62 out of 89 sequenced essential genes in the chromosome III and chromosome V balanced regions. The majority of genes with only one allele identified on chromosome III had secondary support by lethal alleles from other laboratories, published RNAi analysis, or PCR results (Table 2 and Table S1 in File S1). In the case of *let-786*, sequencing led to two candidate genes: *prp-8* and *unc-116*. Two alleles of *let-712* (*s2439* and *s2598*) are in *prp-8*. Both *let-712* alleles complement *let-786*(*s2631*), therefore *let-786* is not *prp-8*. *unc-116*(*rh24*) is lethal, therefore *unc-116* is a lethal gene and we concluded that *unc-116* and *let-786* are likely the same gene.

**Table 2:** The number of essential genes identified in this study

Essential Genes Sequenced	Allele Sequenced	Genes Identified	Total Essential Genes Identified
*Chromosome III* (mid/*sDp3*): 43 genes	37 genes with single allele sequenced	21 of 37 genes identified	27 of 43 genes
	Six genes with two alleles sequenced	Six of six genes identified	
*Chromosome V* (left/eT*1*): 46 genes	Six genes with single allele sequenced	Five of six genes identified	35 of 46 genes
	40 genes with two alleles sequenced	30 of 40 genes identified	
Total: 89 genes			62 (60 mapped molecularly)

Fourteen genes were confirmed by experiments using PCR sequencing, rescue assay, and complementation tests. In the chromosome III and chromosome V balanced regions, 37 and 4, respectively, have only one Illumina sequenced allele. For five genes on III and four on V, there were multiple variations in different candidate genes in the zones they were mapped to. To test the candidate genes, we took advantage of the second alleles of those genes (Johnsen and Baillie 1991; Stewart *et al.* 1998). We PCR-amplified the candidate genes using genomic DNA of a strain with the second allele, which was followed by Sanger sequencing both ends of the PCR fragments. Using this method, we were able to identify five genes on chromosome III and four on chromosome V.

**Figure 2:**
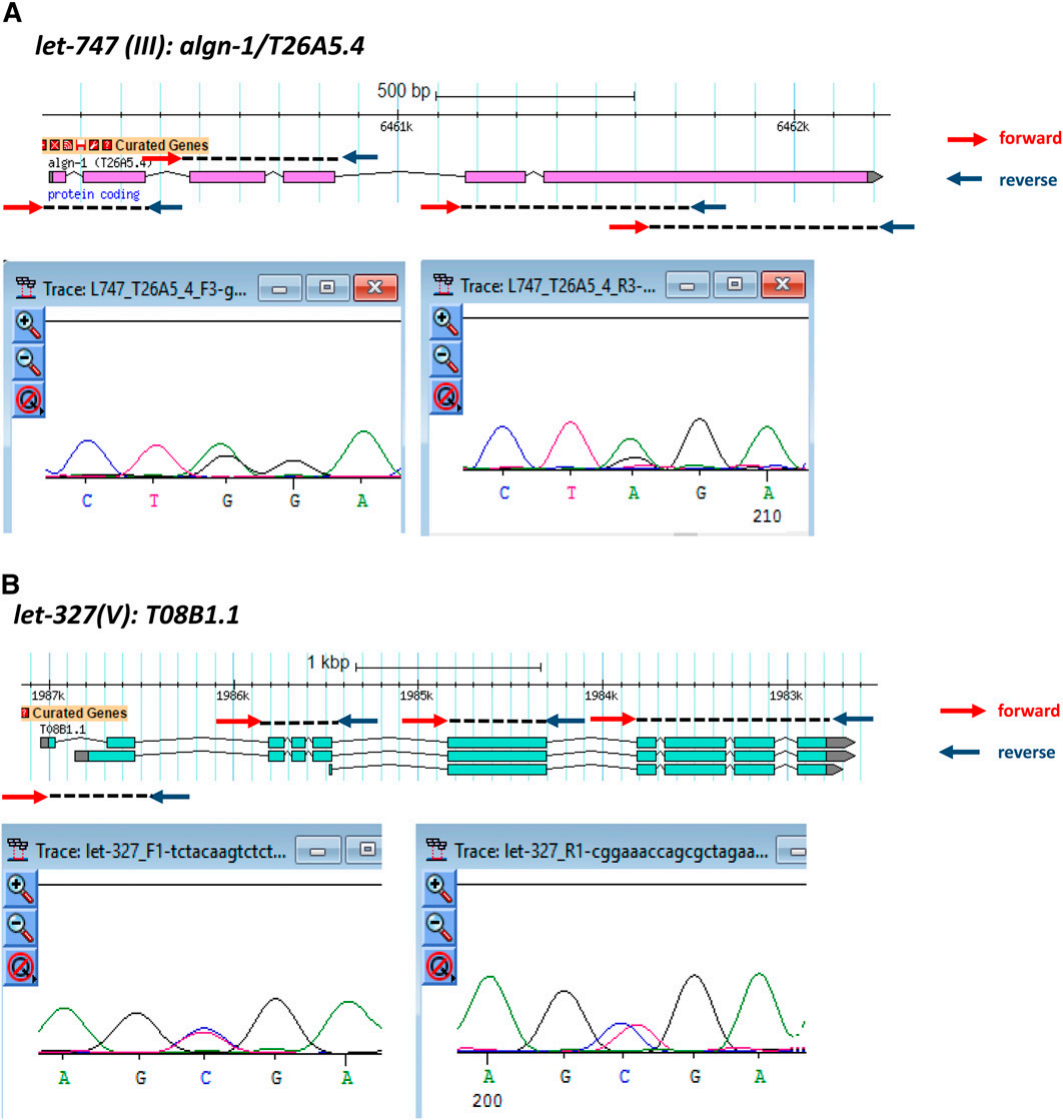

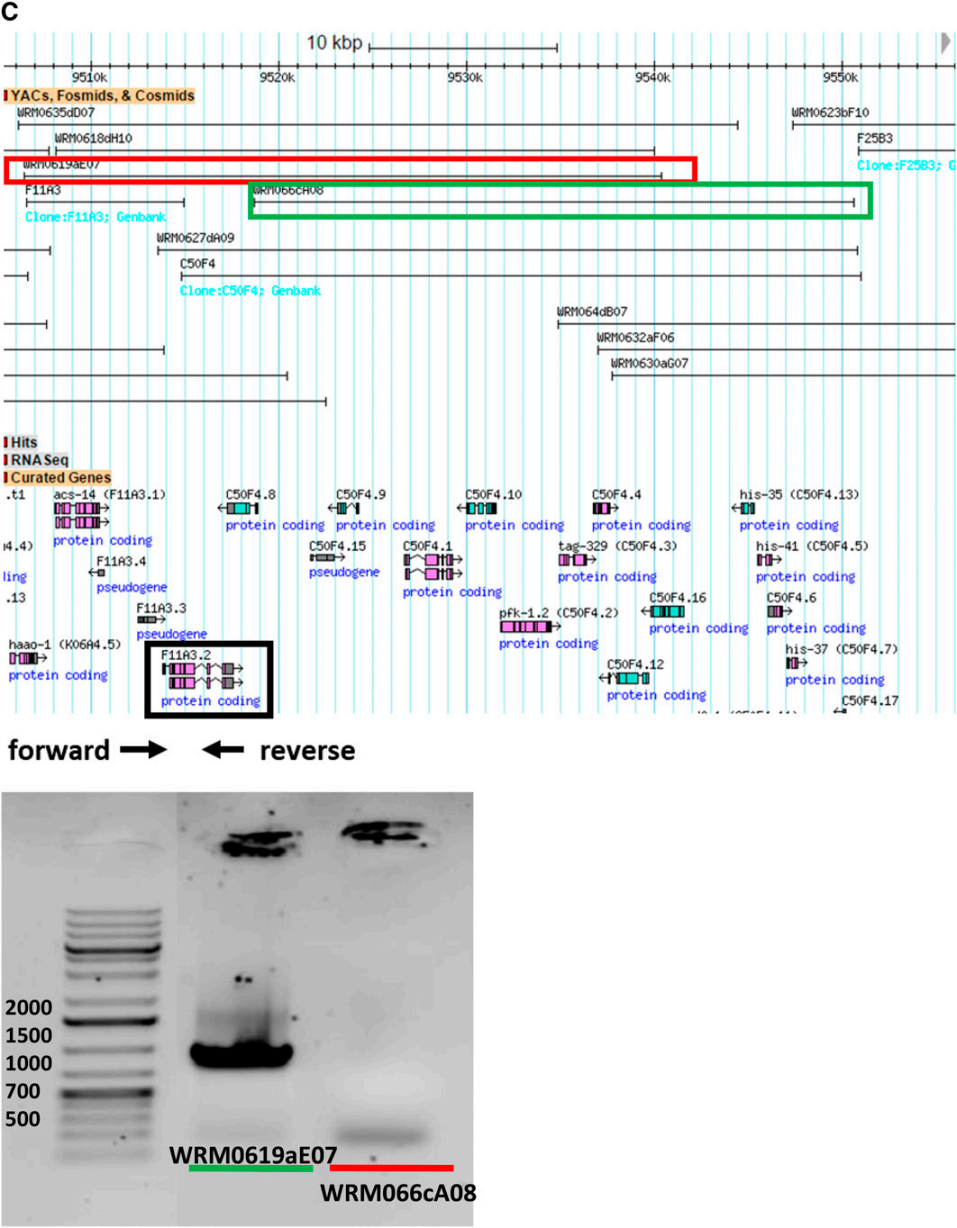
Three examples of PCR sequencing validation and rescue assay. (A) Gene validation of *algn-1/T26AA5.4* for *let-747* on chromosome III. (B) Gene validation of *T08B1.1* for *let-327* on chromosome V. The gene model in each figure shows that the designed primers cover all coding exons of each gene. The red arrows are forward primers while the arrows in dark blue are reverse primers. Under each gene model, there are partials of Sanger sequencing results using forward and reverse PCR primers as sequencing primers. For *let-747/algn-1/T26AA5.4*, a point mutation (G->A) was found in the last exon causing a premature stop codon. A C->T point mutation was found in the second exon of *T08B1.1a* and the first exon of *T08B1.1b*. A premature stop codon occurred in both *T08B1.1* transcripts because of this mutation. (C) Fosmids used for the rescue assay. The fosmid (*WRM0619aE07*) containing the candidate gene, *F11A3.2*, is in the red box while *WRM066cA08* without *F11A3.2* is in the green box. *F11A3.2* is in the black box with the designed forward primer and reverse primer underneath. The gel image shows the presence of *F11A3.2* in *WRM0619aE07* with a target band of 794 bp and the absence of *F11A3.2* in *WRM066cA08* without the 794 bp PCR product. The weak band of ∼200 bp might be a nonspecific band because it can be found in both products using two fosmids as template.

Figure 2 includes two examples of validated genes, one from chromosome III and the other from chromosome V, which were successfully validated. Due to the heterozygosity of the lethal mutation balanced by duplications or translocations, one would expect to see overlapping sequencing peaks for both the wild-type allele and mutation allele at the same position. Because duplication balancers cover two lethal alleles over one wild-type allele, we see the height of the sequence peak of the mutant allele is slightly higher than the wild-type allele. However, the displayed nucleotide can be the wild-type allele or the mutant allele when Sanger sequencing was performed from both ends of the PCR products (Figure 2A). For translocations, the ratio of mutant allele to wild-type allele is 1:1, therefore the sequencing peaks from the two alleles have identical heights. However, the displayed nucleotide for *let-327* is the wild-type allele when sequenced from both strands (Figure 2B). In either case, considering that the relative abundances of the wild-type allele and mutated allele are not easily distinguishable in heterozygotes, the lethal mutations could be easily missed.

No mutation was identified in the zones containing *let-470*(*s1581*) and *let-470*(*s1629*). However, *let-470*(*s1581*) has only *F11A3.2* as a candidate essential gene, with a splicing acceptor mutation besides the zone boundary. To test if *F11A3.2* is *let-470*(*s1629*), we performed rescue assay using two different fosmids. The result showed that the fosmids containing the wild-type copy of *F11A3.2* rescues the lethal phenotype of *let-470*(*s1629*) while the fosmids without the candidate gene did not rescue the lethal phenotype. Figure 2C shows the genomic locations of two fosmids that were used for the rescue assay. PCR was also conducted to confirm the presence or absence of the candidate gene in the two fosmids.

Complementation tests were carried out on four genes. *let-459*(*s1615*) contains a mutation that changes the splicing signal, causing a frame shift and premature stop codon in *hpo-18/F32D1.2*. WGS analysis did not find a corroborating mutation in the *let-459*(*s1432*) sequence. A knockout allele *hpo-18*(*ok3436*) is lethal. We did complementation tests between *hpo-18*(*ok3436*) and both *let-459*(*s1432*) and *let-459*(*1615*). Both tests showed failure to complement, which confirmed that *hpo-18* and *let-459* are the same gene. Complementation tests also showed that *let-447* and *egl-8* are the same gene. The phenotype of *egl-8*(*md1971*) includes reduced locomotion, reduced body flexion at rest, and loopy backing. Both *let-447*(*s1664*)*/egl-8*(*md1971*) and *let-447*(*s1457*)*/egl-8*(*md1971*) have the *egl-8*(*md1971*) phenotype, and therefore failure to complement. Strains used for complementation tests are summarized in Table S4 in File S1.

Complementation tests were used to recheck genes whose mutations were found in genes with different “let” names. *let-439* has two sequenced alleles and both of them have mutations in *M03F8.3*, showing that it is *let-439*. However, *let-443*(*s1417*) had previously been mapped to a different zone than *let-439*, but contained a nonsense mutation in *M03F8.3*. We complementation-tested *let-443*(*s1417*) against both alleles of *let-439* and they failed to complement, which supported the sequencing result; therefore, we renamed *let-443*(*s1417*) *let-439*(*s1417*). Complementation tests also showed that both *let-757*(*s2460*) *and let-757*(*s2867*) fail to complement *let-821*(*s2804*) and sequence analysis showed them to be *fbn-1* (*ZK783.1*). These two results reduce the number of genes from 62 to 60, so 60 identified genes have been mapped to their molecular counterpart (Table 2). In addition, a total of 104 lethal mutations (from 135 sequenced strains) were molecularly discovered in 60 essential genes with 41 missense mutations and 39 nonsense mutations (Table S1 in File S1). We did not identify any lethal mutations in the other 31 sequenced strains.

All these cases show the value of combined genetic and sequencing analyses because they not only help with narrowing down the search region for lethal mutations, but also aid in correcting and improving the quality of genetic mapping.

We checked the 60 genes on WormBase for lethal alleles (including gk, tm, and ok alleles) that were presented in previous publications. We summarized the genes into three categories and details of each gene are listed in the last column of Table S1 in File S1. The first category is genes published with lethal alleles (*n* = 12; two with only tm alleles, these are highlighted in green). Five of those genes, *unc-116*, *lin-13*, *egl-8*, *sos-1*, and *mig-6*, were published prior to Johnsen and Baillie (1991) and Stewart *et al.* (1998). See Stewart *et al*. (1998) for *let-786* and *let-752* and Johnsen and Baillie (1991) for *let-447*, *let-344* and *let-423*. Therefore, *let-786* is *unc-116*, *let-752* is *lin-13*, *let-447* is *egl-8*, *let-344 is sos-1*, and *let-423* is *mig-6*. The other seven were first published in Stewart *et al.* (1998) and Johnsen and Baillie (1991) and therefore the “let” names in those publications take precedent. The second category is genes that have known lethal alleles that have not been curated and characterized (*n* = 18; all of the alleles listed are gk, tm, or ok; these are black in the table. Their correct names were first published in Stewart *et al.* (1998) or Johnsen and Baillie (1991). The third category is genes that have no alleles in WormBase (*n* = 30; highlighted in red in the table). Their gene names are published in Stewart *et al.* (1998) or Johnsen and Baillie (1991).

### Functions of the identified 60 essential genes

We looked at the functions of the 60 essential genes that we identified. Most of these genes, based on orthologous relationships, have biological functions that have been predicted in other organisms. Among the 60 essential genes, 22 encode enzymes, including helicases, kinases, and tRNA synthetases. There are also structure proteins including ribosomal proteins and transcription factors, as well as mRNA splicing factors. Note that seven of our essential genes have no known functions. Five of these seven genes have orthologs in nematodes but no other organisms.

We took advantage of WormBase to investigate the evolutionary conservation of each gene by looking for orthologs in Nematodes (N), Invertebrate (*D. melanogaster*) (I), Mammals (mouse as well as human) (M), and single-cell Fungi (*Saccharomycetaceae*) (F), as shown in Table 3.

**Table 3:** Biological functions of the 60 essential genes

*let*-Name	Chr	Gene Name	Protein Function	Pathway	Evolutionary Conservation
*let-727*	III	*R02F2.7*	Unknown	Unknown	N
*let-728*	III	*C23G10.8*	Unknown	Unknown	N
*let-752*	III	*lin-13*	Zinc-regulated transcription factor	In *C. elegans*, LIN-13 is involved in the tumor suppressor Rb-mediated transcriptional control process that leads to repression of vulval fates.	N
*let-798*	III	*wrm-1*	*C. elegans* β-catenin-like protein	In *C. elegans*, wrm-1 functions in noncanonical Wnt signaling pathways that specify cell fates in the early embryo.	N
*let-342*	V	*pmt-2*	Enzyme: phosphoethanolamine N-methyltransferase	PMT-2 lacks known mammalian orthologs, but has orthologs in the parasitic nematodes: fish, plants, and bacteria. PMT-1 only catalyzes the conversion of phosphoethanolamine to phospho-monomethylethanolamine, which is the first step in the PEAMT-pathway.	N
*let-428*	V	*K03B4.1*	Unknown	Unknown	N
*let-455*	V	*Y45G5AM.9*	Unknown	Unknown	N
*let-463*	V	*C04E6.11*	Unknown	Unknown	N
*let-757/let-821*	III	*fbn-1*	Extracellular matrix protein fibrillin	Fibrillin is a glycoprotein, which is essential for the formation of elastic fibers found in connective tissues.	N, I, M
*let-782*	III	*tag-189*	Unknown	Unknown	N, I, M
*let-827*	III	*cee-1*	Unknown	Unknown	N, I, M
*let-327*	V	*T08B1.1*	Member of solute carriers family	Predicted to have transmembrane transporter activity.	N, I, M
*let-344*	V	*sos-1*	Son of sevenless homolog	Its ortholog in mouse is a catalytic component of a trimeric complex that participates in transduction of signals from Ras to Rac by promoting the Rac-specific guanine nucleotide exchange factor activity. SOS-1 is involved in multiple Ras-dependent signaling pathways, which also interacts with LET-23 and LET-60 during vulval development.	N, I, M
*let-348*	V	*rft-1*	Member of solute carriers family, riboflavin transporter	In *C. elegans*, *rft-1* exhibits riboflavin transporter activity and is involved in embryo development and receptor-mediated endocytosis.	N, I, M
*let-423*	V	*mig-6*	Highly similar to the extracellular matrix proteins papilin and lacunin	In *C. elegans*, MIG-6 activity is required for the ventral to dorsal phase of distal tip cell migration.	N, I, M
*let-440*	V	*ncx-2*	Sodium/calcium exchangers	Mediates the electrogenic exchange of Ca^2+^ against Na^+^ ions across the cell membrane, thereby contributes to the regulation of cytoplasmic Ca^2+^ levels and Ca^2+^-dependent cellular processes.	N, I, M
*let-702*	III	*hmgr-1*	Enzyme: 3-hydroxy-3-methylglutaryl-coenzyme A reductase	HMG-CoA reductase catalyzes the conversion of HMG-CoA to mevalonate, which is a rate-limiting step in sterol biosynthesis.	N, I, M, F
*let-712*	III	*prp-8*	mRNA splicing: pre-mRNA-processing-splicing factor	Its ortholog in yeast is a component of the U4/U6-U5 snRNP complex and participates in spliceosomal assembly through its interaction with the U1 snRNA.	N, I, M, F
*let-732*	III	*rpb-2*	Enzyme: RNA polymerase II (B) subunit	The second largest subunit B150 of RNA polymerase II is the enzyme that produces the primary transcript.	N, I, M, F
*let-736*	III	*cdk-12*	Enzyme: cyclin-dependent kinase	Its ortholog in yeast phosphorylates both the RNA pol II subunit to affect transcription, and the ribosomal protein to increase translational fidelity.	N, I, M, F
*let-741*	III	*rars-1*	Enzyme: arginyl(R) aminoacyl tRNA synthetase in mitochondria	The tRNA synthetase catalyzes the attachment of an amino acid to its cognate transfer RNA molecule.	N, I, M, F
*let-743*	III	*ZK686.2*	Enzyme: ATP-dependent RNA helicase	Its orthologs in yeast and human are involved in the biogenesis of ribosomal subunits.	N, I, M, F
*let-747*	III	*algn-1*	Enzyme: chitobiosyldiphosphodolichol β-mannosyltransferase	Mannosyltransferase is involved in asparagine-linked glycosylation in the endoplasmic reticulum.	N, I, M, F
*let-753*	III	*C34E10.10*	Ribosome-related protein: rRNA-processing protein	The protein is involved in ribosomal subunit biogenesis.	N, I, M, F
*let-763*	III	*T08A11.2*	mRNA splicing: splicing factor 3B subunit 1	U2-snRNP associated splicing factor forms extensive associations with the branch site-3′ splice site-3′ exon region upon prespliceosome formation.	N, I, M, F
*let-764*	III	*byn-1*	Mammalian bystin (adhesion protein) related protein	Its ortholog in yeast is required for pre-rRNA processing and 40S ribosomal subunit synthesis.	N, I, M, F
*let-771*	III	*rfl-1*	Enzyme: ubiquitin activating enzyme	RFL-1 activity is required for proper cytokinesis and spindle orientation.	N, I, M, F
*let-774*	III	*rps-3*	Ribosome-related protein: ribosomal protein, small subunit	Protein component of the small ribosomal subunit involved in protein biosynthesis.	N, I, M, F
*let-784*	III	*gop-3*	Component of the Sorting and Assembly Machinery (SAM) complex	SAM complex binds precursors of beta-barrel proteins and facilitates their outer membrane insertion.	N, I, M, F
*let-786*	III	*unc-116*	Kinesin-related motor protein	Its ortholog in yeast is required for mitotic spindle assembly and chromosome segregation.	N, I, M, F
*let-799*	III	*ddx-23*	Enzyme: ATP-dependent RNA helicase	Its ortholog in yeast is involved in mRNA decay and rRNA processing.	N, I, M, F
*let-826*	III	*mrps-18C*	Ribosome-related protein: ribosomal protein (small) in mitochondria	Ribosomal subunit biogenesis	N, I, M, F
*let-829*	III	*atp-2*	Enzyme: mitochondrial ATP synthase subunit	Evolutionarily conserved enzyme complex that is required for ATP synthesis.	N, I, M, F
*let-832*	III	*F09F7.4*	Enzyme: 3-hydroxyisobutyryl-CoA hydrolase	Biological process unknown	N, I, M, F
*let-972*	III	*hsp-110*	Heat shock protein	Its ortholog in yeast is a ATPase component of the heat shock protein Hsp90 chaperone complex and serves as nucleotide exchange factor to load ATP onto the SSA class of cytosolic Hsp70s.	N, I, M, F
*let-326*	V	*rab-1*	Enzyme: Rab family GTPase	Intracellular vesicle trafficking, such as the ER-to-Golgi step of the secretory pathway.	N, I, M, F
*let-331*	V	*prx-6*	Peroxisomal biogenesis factor	Its ortholog in yeast heterodimerizes with Pex1p and participates in the recycling of the peroxisomal signal receptor from the peroxisomal membrane to the cystosol.	N, I, M, F
*let-332*	V	*C05C8.7*	Enzyme: Mannose phosphate isomerase	Its ortholog in yeast catalyzes the interconversion of fructose-6-P and mannose-6-P, which is required for early steps in protein mannosylation.	N, I, M, F
*let-334*	V	*slc-17.8*	Member of solute carriers family	Predicted to have transmembrane transporter activity.	N, I, M, F
*let-335*	V	*C37C3.2*	Translation initiation factor	Based on the homology to yeast, the products of C37C3.2 are predicted to function during translation initiation as GTPase activators to stimulate GTP hydrolysis by eIF2-GTP-Met-tRNA_i_.	N, I, M, F
*let-338*	V	*rpac-40*	Enzyme: RNA polymerase I/III (A/C) shared subunit	Common component of RNA polymerases I and III, which synthesize ribosomal RNA precursors and small RNAs, such as 5S rRNA and tRNAs.	N, I, M, F
*let-343*	V	*cpsf-2*	Cleavage and polyadenylation specificity factor	Required for 3′ processing, splicing, and transcriptional termination of mRNAs and snoRNAs.	N, I, M, F
*let-346*	V	*soap-1*	HEAT repeat-containing protein	SOAP is involved in a pathway that controls the apical delivery of E-cad and morphogenesis.	N, I, M, F
*let-350*	V	*C37H5.5*	Nucleolar complex protein-like DNA replication regulator	Its ortholog in yeast binds to chromatin at active replication origins and is required for pre-RC formation as well as maintenance during DNA replication licensing.	N, I, M, F
*let-402*	V	*erfa-1*	Translation termination factor	Subunit of the heterodimeric translation release factor complex involved in the release of nascent polypeptides from ribosomes.	N, I, M, F
*let-408*	V	*snap-1*	α-soluble NSF attachment protein	Its ortholog in mouse is required for vesicular transport between the endoplasmic reticulum and the Golgi apparatus.	N, I, M, F
*let-409*	V	*asns-1*	Enzyme: asparagine synthase (glutamine-hydrolyzing)	Catalyzes the synthesis of L-asparagine from L-aspartate in the asparagine biosynthetic pathway.	N, I, M, F
*let-410*	V	*dlst-1*	Enzyme: dihydrolipoamide S-succinyltransferase	Its ortholog in yeast is a component of the mitochondrial alpha-ketoglutarate dehydrogenase complex, which catalyzes the oxidative decarboxylation of alpha-ketoglutarate to succinyl-CoA in the TCA cycle.	N, I, M, F
*let-411*	V	*xpo-1*	Exportin-1, an importin-β-like protein orthologous to *Drosophila*, vertebrates, and yeast exportin-1/CRM1	XPO-1 is predicted to function as a nuclear export receptor for proteins containing leucine-rich nuclear export signals.	N, I, M, F
*let-415*	V	*hsp-6*	Nuclear-encoded mitochondrion-specific chaperone that is a member of the DnaK/Hsp70 superfamily of molecular chaperones	In *C. elegans*, *hsp-6* is involved in the mitochondrial unfolded protein response.	N, I, M, F
*let-417*	V	*ceh-34*	Homeobox protein	In *C. elegans*, *ceh-34* activity is required for regulation of the programmed cell death of a pharyngeal neuron, the sister of the M4 motor neuron.	N, I, M, F
*let-419*	V	*pqn-51*	Transcription initiation factor IIA	Its ortholog in yeast is involved in transcriptional activation and acts as antirepressor or coactivator.	N, I, M, F
*let-420*	V	*adss-1*	Enzyme: adenylosuccinate synthetase	Catalyzes the first step in the synthesis of adenosine monophosphate from inosine 5′monophosphate during purine nucleotide biosynthesis.	N, I, M, F
*let-422*	V	*hmgs-1*	Enzyme: hydroxymethylglutaryl-CoA synthase	Its ortholog in yeast catalyzes the formation of HMG-CoA from acetyl-CoA and acetoacetyl-CoA.	N, I, M, F
*let-424*	V	*vars-1*	Enzyme: valyl-aminoacyl tRNA synthetase in mitochondria	The tRNA synthetase catalyzes the attachment of an amino acid to its cognate transfer RNA molecule.	N, I, M, F
*let-439*	V	*M03F8.3*	mRNA splicing: crooked neck pre-mRNA splicing factor	The crooked neck gene of *Drosophila* is essential for embryogenesis and is thought to be involved in cell cycle progression and pre-mRNA splicing.	N, I, M, F
*let-442*	V	*C05C8.2*	Ribosome-related protein: small subunit processome component	A nucleolar protein required for rRNA synthesis and ribosomal assembly.	N, I, M, F
*let-447*	V	*egl-8*	Enzyme: phospholipase C β	In yeast, the ortholog (Plc1p) and inositol polyphosphates are required for acetyl-CoA homeostasis, which regulates global histone acetylation.	N, I, M, F
*let-459*	V	*hpo-18*	Enzyme: ATP synthase	Unknown	N, I, M, F
*let-470*	V	*F11A3.2*	Translation initiation factor	These proteins help stabilize the formation of the functional ribosome around the start codon and also provide regulatory mechanisms in translation initiation.	N, I, M, F

The table is sorted by evolutionary conservation, chromosome (Chr), and lethal name (*let*-Name). N, nematodes; I, invertebrates (*Drosophila*); M, mammals (mouse, human); F, fungi (*Saccharomycetaceae*).

Because of the functional importance of essential genes, one would expect that they are conserved and found in most organisms. Consistent with this, we found that 44 out of 60 (73.3%) of our essential genes have orthologs in all the examined organisms but eight of the 60 (13.3%) are nematode-specific. This is consistent with the results found in a previous study based on a transcriptome analysis of the phylum Nematoda (Parkinson *et al.* 2004). Eight genes were found in nematodes, invertebrates, and mammals, but not in single-cell fungi, suggesting that there are essential genes that are specific to multicellular organisms. For instance, *let-757/fbn-1* encodes the extracellular matrix protein fibrillin, which is highly conserved in multicellular organisms and is a key component of elastic fiber in connective tissues (Ritty *et al.* 2003).

### Four new protein domains revealed by examining missense lethal mutations

Protein functions can be affected by missense mutations that reside in conserved regions, such as functional domains. Thus, we want to explore whether missense lethal mutations are more likely to be located within annotated Pfam domains.

**Figure 3:**
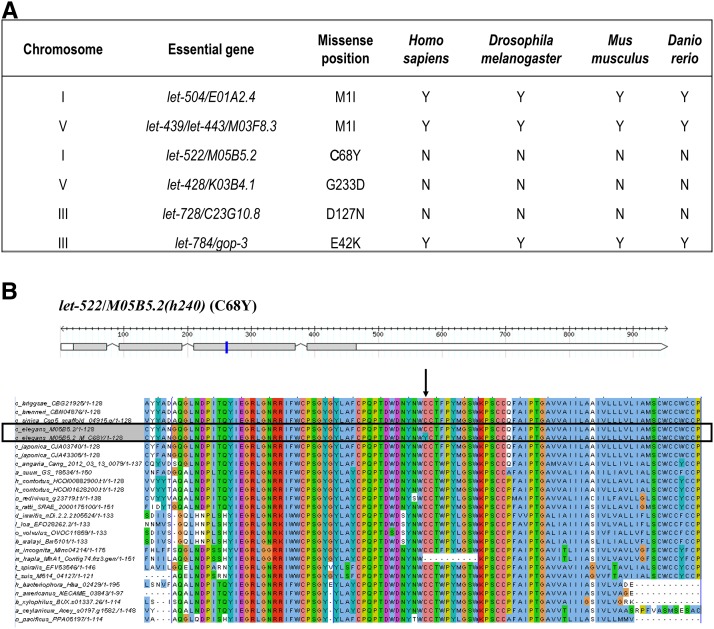
(A) Six missense mutations in six essential genes that are not in annotated functional domains. (B) One gene model (*let-522/M05B5.2*) that has a missense mutation *h240* (C68Y) that does not reside in an annotated domain (Chu *et al.* 2014). The gene model was created using the Perl module FeatureStack. Exons are drawn to scale relative to each other in gray boxes; black lines with triangle shapes represent introns, which are not drawn to scale; white boxes represent UTRs. Blue bars indicate the missense mutation. Multiple alignments were performed between the essential genes and their orthologs in 25 other nematodes and four model organisms (*Homo sapiens*, *Drosophila melanogaster*, *Mus musculus*, and *Danio rerio*). However, no orthologs were found in the four model organisms. Left columns show the names of nematode species. The alignment figure shows a fragment of 100 amino acids. The mutated amino acid (C68Y) as indicated by the black arrow is in the center with 50 amino acids upstream and 49 amino acids downstream. The alignment color scheme is based on “ClustalX,” in which the color of a symbol depends on the residue type and the occurrence frequency in one column. Black boxes highlight the input genes with both wild type and mutant copy.

To gain a better understanding of lethal mutations in *C. elegans*, we wanted a large dataset and so incorporated 62 essential genes identified in previous studies that examined lethal mutations balanced by *sDp2* on chromosome I (Chu *et al.* 2014) and *eT1* on chromosome V (Jones *et al.* 2007), and the essential genes identified in this study. We found 70 missense mutations in 59 essential genes of which 64 are within Pfam domains of 53 genes, suggesting the functions of the essential proteins are disrupted. We looked at the remaining six missense mutations in six essential genes (Figure 3A).

Two missense mutations were nucleotide transitions (G->A) that changed the ATG start codon to ATA. It is possible that the next in-frame ATG would be recognized as an initiation codon, but the truncated product could have a hypomorphic defect (Maser *et al.* 2001). In both cases in our study, the next ATG was not in-frame, which probably resulted in a lack of wild-type products causing loss of function (Schnabel *et al.* 1992). The observed lethal phenotypes also show that start codon mutations can be deleterious.

For the other four missense mutations, even though they are not located within annotated domains (by searching InterPro database), it turns out that the altered amino acids are still highly conserved in orthologs from different species. In a window size of 100 amino acids, which is the average size of protein domains based on the statistical analysis on the size of Pfam domains from all protein isoforms (data not shown), the sequence regions containing the target amino acids are also highly conserved (one example is shown in Figure 3B). It suggests new protein domains that had not been identified as functional domains. It is also possible that the effected amino acids are important for maintaining protein structures and the mutations disrupt these structures, resulting in malfunctioning proteins.

### GO analysis of essential genes

To conduct GO analysis, four groups of genes were used for comparison. Group one (G1): essential genes that were isolated through genetic screens in the Baillie laboratory or collaborations with the Baillie laboratory and have been fully sequenced or rescued by fosmids (Maciejowski *et al.* 2005; Jones *et al.* 2007; Chu *et al.* 2014; S. Ono, personal communication) (143 in total, including 60 genes from the current study). Group two (G2): essential genes that have published lethal alleles (1208 in total). Group three (G3): genetically identified nonessential genes (796 in total). Group four (G4): genes with no observed phenotype by either RNAi or alleles (12,811 in total). This putatively nonessential group contains the majority of protein-coding genes in *C. elegans*.

**Figure 4:**
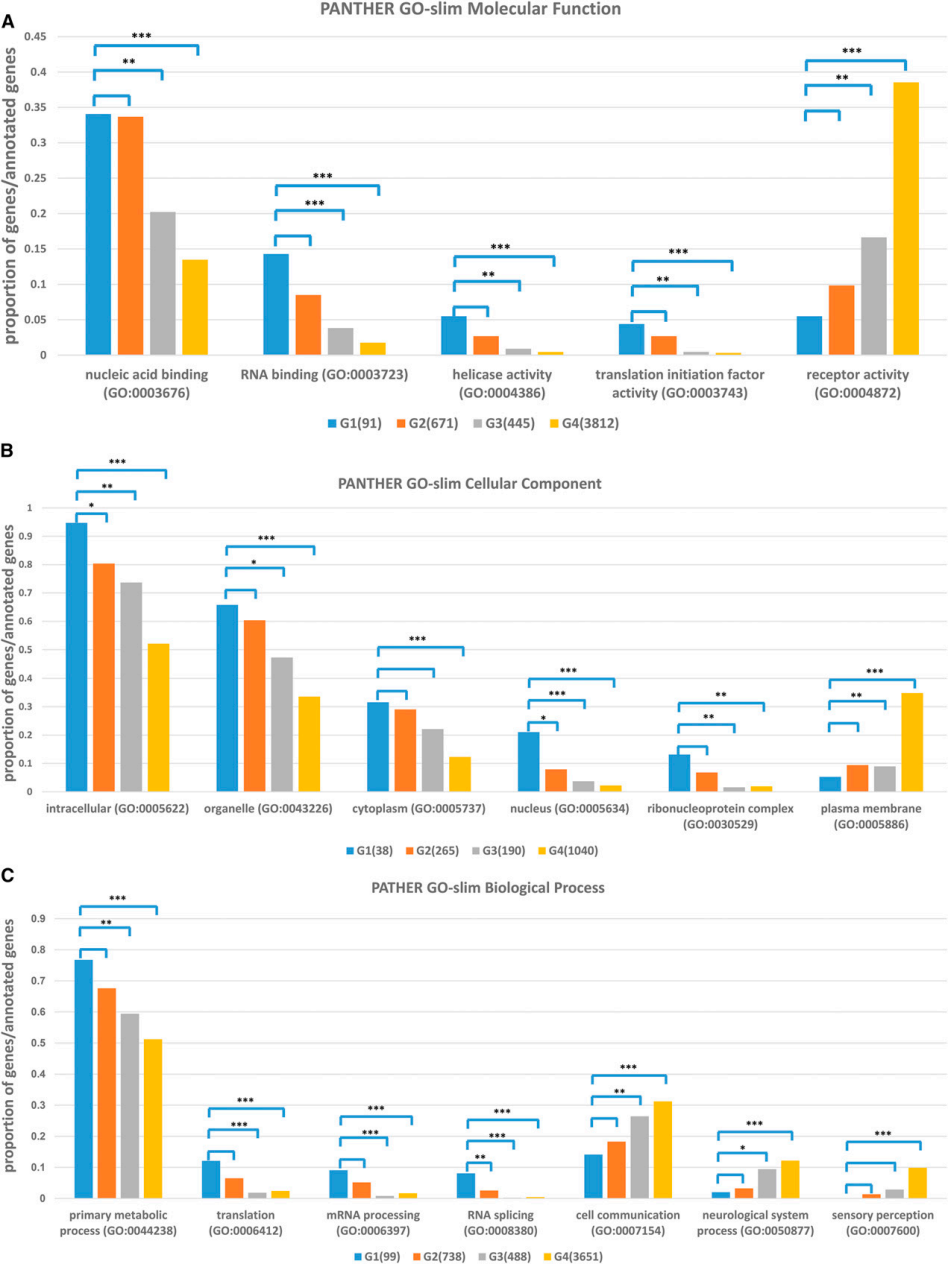
Genes from each group annotated with three ontology terms: (A) molecular function, (B) cellular component, and (C) biological process. The *x*-axis lists several GO terms in each GO category and the *y*-axis is the proportion of genes for each GO term over the total number of annotated genes in each group. Four groups are shown in separate colors: G1 in blue, G2 in orange, G3 in gray, and G4 in gold. Statistical difference was calculated for G1 *vs.* G2, G3, and G4 individually by using Fisher’s exact test (* *P*-value < 0.05, ** *P*-value < 0.01, *** *P*-value < 0.001).

We compared the G1 essential genes to genes in the other three groups based on three GO categories: cellular component, molecular function, and biological process (Figure 4).

Essential proteins from G1 and G2 have no significant difference in any molecular function related annotation, including nucleic acid binding and especially RNA binding (GO:0003723). These annotations are significantly lower for the proteins of nonessential genes for both G3 and G4 (Figure 4A). This is consistent with our observation in the cellular component analysis, in which annotations of the ribonucleoprotein complex (GO:0030529) are high in essential proteins (Figure 4B). In contrast, receptor activity (GO:0004872) is lower in essential proteins (Figure 4A). This might also explain the observation that the proportion of nonessential proteins located in plasma membranes (GO:0005886) is significantly higher than that of essential proteins (Figure 4B).

With regard to biological processes, essential proteins in G1 and G2 are significantly enriched for primary metabolic processes, as well as translation and mRNA processing, suggesting that essential genes tend to be involved in protein synthesis. In contrast, nonessential proteins are significantly enriched for regulation of cellular functions, such as cell communication and sensory perception (Figure 4C). If there is a disruption in these processes, the worms might show visible phenotypes; however, these are generally not lethal.

GO term analysis indicated that essential genes tend to execute enzyme and nucleic acid binding activities during fundamental processes, such as DNA replication, translation, and transcription intracellularly.

### Gene essentiality analysis

Gene essentiality, gene duplicability, protein interaction networks, and gene expression are biological factors that can influence the evolutionary rate of proteins (Liao *et al.* 2006). Thus, to develop an understanding of how essential proteins function in cells, we assessed the properties of essential genes from three perspectives: gene duplicability, protein interactions, and gene expression, applying the same dataset used in GO term analysis.

#### Gene essentiality *vs.* gene duplicability:

**Figure 5:**
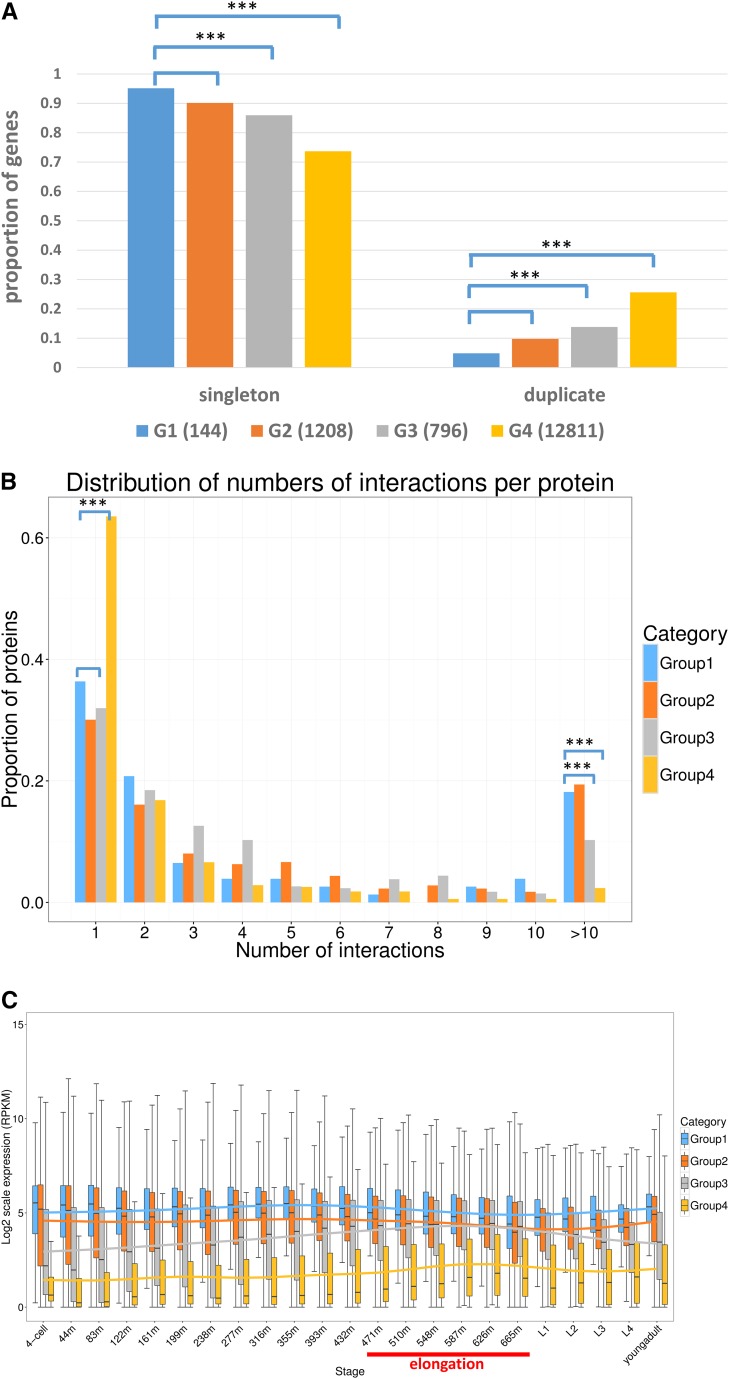
Gene essentiality analysis. Group one (G1): essential genes that were isolated through genetic screens in the Baillie laboratory or collaborations and are fully sequenced or rescued by fosmids (Maciejowski *et al.* 2005; Jones *et al.* 2007; Chu *et al.* 2014; S. Ono, personal communication) (143 in total, including 60 genes from the current study). Group two (G2): essential genes that have published alleles supporting lethal phenotypes (1208 in total). Group three (G3): nonessential genes that have known alleles supporting nonlethal phenotypes (796 in total). Group four (G4): no-phenotype supporting genes that have no observable phenotypes caused by either RNAi or known alleles (12,811 in total). Given that this group contains the majority of protein-coding genes in *C. elegans*, most genes in this group are expected to be nonessential. The four groups are labeled in blue, orange, gray, and gold, respectively. (A) The proportion of singletons and duplicates in the four groups. The *x*-axis shows singletons and duplicates from the groups. Significant differences (*** P-value < 0.001) were observed between G1 and G3, and between G1 and G4. (B) The distribution of number of interactions per protein (Stark *et al.* 2006). The *x*-axis shows the number of interactions per protein and the *y*-axis is the proportion of proteins. (C) The developmental stage-specific expression pattern and expression level of genes in four groups. The *x*-axis shows 23 developmental stages, embryos to young adults, from four cells. m in the *x*-axis is short for minutes of embryonic development. Each bar represents the distribution of the normalized expression level (RPKM in log2 scale) of each gene in each of four groups for each developmental stage. The linear regression trend line is drawn for each group in their respective color. The stages of embryonic elongation are underlined in red. The boxplot was computed using R and the function ggplot2, which uses a 95% C.I.

In *C. elegans*, researchers showed that closely related gene duplicates are responsible for mitigating the effects of mutations (Conant and Wagner 2004), which is also known as mutational robustness (Mendonca *et al.* 2011). Studies performed in yeast showed that duplicate genes have a higher probability of functional compensation than singletons (Gu *et al.* 2003). However, there are analyses in both yeast and mouse suggesting that duplicate genes are as essential as singletons (Wagner 2000; Liang and Li 2007; Liao and Zhang 2007). Thus, we want to revisit this question using our collected datasets. Using the most stringent criteria of *e*-value with 1E−20 for quantitative comparison, we see that most collected genes are singletons, no matter which group they belong to (Figure 5A). This is consistent with the theory that redundant duplicates are not evolutionarily stable and tend to be removed over long periods of time (Nowak *et al.* 1997), although this theory is still debated (Yona *et al.* 2015). Despite that, the proportions of singletons to duplicates in each group show differences. The proportions of both singletons and duplicates in G1 are similar to those in G2, which is as we expected, considering both groups contain only essential genes. On the other hand, the proportions of singletons are significantly less in non-essential genes (both G3 and G4). Based on this analysis, essential genes are more likely to be unique, with over 90% of essential genes present as single copies (in both G1 and G2) compared with nonessential genes.

#### Gene essentiality *vs.* gene connectivity:

Proteins do not function alone; they function as parts of pathways, macromolecular structures, and regulatory networks (Dolinski and Botstein 2007). By characterizing the yeast proteome, Jeong *et al.* (2001) found that the most highly connected proteins in a cell are the most important ones for survival (Jeong *et al.* 2001). Based on this theory, we hypothesize that essential proteins tend to engage in protein interaction hubs, with more interactors connecting to them than to nonessential proteins.

Using the whole genome protein interactions in *C. elegans* from BioGRID (Stark *et al.* 2006), Figure 5B shows the distribution of the number of interactions per protein, essential proteins from G1 and G2 tend to have more interaction partners, *i.e.*, >10 proteins compared with G3 and G4 (*P*-value < 0.001). This suggests that essential proteins tend to be in the interaction hubs. It is interesting to note that proteins that have no phenotypic consequence are much more likely to have only one interactor.

#### Gene essentiality *vs.* gene expression:

Expression levels are frequently used to infer the activities and functions of gene products. Expression levels have also been used as a parameter to analyze the nonessential gene datasets in the human genome (Hart *et al.* 2014). Therefore, we want to examine the expression level of all *C. elegans* genes in our collected data by using the gene expression dataset from the *GExplore* (version 1.4) expression search interface (Hutter *et al.* 2009) and the NHGRI modENCODE project (Hillier *et al.* 2009; Gerstein *et al.* 2010).

Figure 5C shows that essential genes from G1 and G2 have significantly higher expression level in all developmental stages than nonessential genes from G3 and G4. With respect to the expression pattern over the developmental stages, essential genes are more consistently expressed across all developmental stages than nonessential genes. However, the expression levels of nonessential genes show a twofold increase in the embryonic elongation stage (470–640 min). During this period, morphogenesis is happening, which in more complex multicellular organisms could be considered as the assembly of cells into functioning tissues and organs. Morphogenesis likely proceeds via conserved signaling pathways, regulatory mechanisms, and effector genes (Edgar 1999) (Figure 5C).

## Discussion

A total of 62 out of 89 essential genes were successfully identified and 60 out of 62 were matched to their molecular counterpart. This large number demonstrates the value of our method. However, we were not able to identify any appropriate mutations in the sequences of the other 27 genes. Complementation tests and PCR sequencing were conducted to validate possible candidates for seven of those 27; however, we found that they were not essential genes. We do not know the reason(s) for this, but we hypothesize several possible reasons. First is strain mix-ups; however, this is unlikely because during the construction of the strains containing *let-500* the terminal phenotypes of all the lethals were the same as noted when the lethals were first analyzed. Second is sequencing errors, as it is possible that there were not enough sequencing reads to support some of the lethal mutations. For example, only one out of 32 sequencing reads (3%) supports C to T change in *unc-46*(*e177*) in the *let-417*(*s204*) strain, but in the remaining 78 sequenced mutants with *unc-46*(*e177*), the variation frequencies of *e177* range from 23 to 78%. This reason is not adequate because most of the time there was sufficient read support to get good results. Third, mutations may have been mismapped. An example is *let-443* and *let-439*, which were mapped genetically into two different zones. Sequence analysis showed they were actually the same gene. This reason is weak because this result shows that we can find mutations in different zones but careful scanning did not find mutations in any zone for the unsolved genes. Fourth, mutations may reside in noncoding regions. An example is *let-470* where no mutation was found in its genetic zone for *s1629* but a splicing acceptor mutation in *F11A3.2* was found for *s1581* by a zone boundary, and fosmid rescue assays showed that *F11A3.2* rescues *let-470*(*s1629*). This reason does not explain why we found a mutation in noncoding DNA for one allele but not for the other. As stated above, we do not have a good reason to explain why 27 genes were not identified, but we are pleased that 62 were identified, with 60 of the 62 mapped to their molecular counterparts.

Of the 60 identified essential genes, five are without RNAi evidence supporting their lethal phenotype (Table S1 in File S1). This finding is meaningful because it indicates that although RNAi is an efficient method for essential gene identification, it does miss roughly 8.3% (five out of 60) of the genes that can be isolated in mutagenesis screens.

Among the 143 essential genes collected in this study, we found 15 genes related to cell division and chromosome partitioning; surprisingly, 11 of those are on chromosome I. However, there was no other functional clustering of genes observed on chromosome III or V. Nevertheless, chemoreceptor gene families are unusually enriched on chromosome V, especially on the chromosomal arms, which might be responsible for signal transduction in *C. elegans* (Robertson and Thomas 2006). Members of this large gene family are not essential genes but inhabit a lot of chromosome V(left), which is consistent with the fact that chromosome V(left) has few essential genes relative to the other autosomes and has a high degree of gene duplications (Kamath *et al.* 2003). The concentration of functionally related genes on chromosomes in a nonrandom pattern is important for understanding how genomes function and evolved. Besides, it may have clinical significance in understanding or predicting disease-causing. clustered genes (Andrews *et al.* 2015).

We noticed that nonsense mutations are significantly enriched relative to missense mutations over duplication and translocation balancers (data not shown). Considering the heterozygosity background for each mutation, we propose that it might be due to the different functional effects of missense mutations and nonsense mutations in heterozygotes. Missense alleles might confer negative interaction with the wild-type allele in heterozygotes by being located in protein functional domains or the subunit interfaces, causing selection against these heterozygotes during genetic screens (Phillips *et al.* 1995; Thomas *et al.* 1997). On the other hand, the nonsense-mediated mRNA decay (NMD) surveillance system degrades nonsense allele products, which suggests that almost no interfering nonsense mutant products are produced, therefore there is no such negative interaction using either duplication or translocation balancers. Consistent with this theory, we found that nonsense mutations are located toward N-terminal protein sequences. To test our hypothesis, one could utilize NMD defective mutants that harbor a genetically balanced strain to isolate lethal mutations. One would expect that the selective enrichment of nonsense alleles would be reduced in a NMD mutant background, because the amount of truncated protein would be greater in the defective NMD background.

This allele-dependent property of essential genes is related to functional effects causing human diseases. Human orthologs of mouse essential genes are linked to numerous human diseases that affect a variety of physiological systems. It has been shown that mutations in human orthologs of embryonic lethal phenotypes, caused by loss-of-function essential mouse genes, can cause lethality in human pregnancies and may result in spontaneous abortions (Goh *et al.* 2007). However, phenotypic symptoms induced by defective essential genes are not restricted to developmental abnormalities, but can also contribute to adult diseases. Unlike null alleles that cause loss of function, some point mutations in these genes do not necessarily lead to a complete loss of protein function (Dickerson *et al.* 2011). There are orthologs in human of mouse essential genes with missense mutations that may lead to abnormal phenotypes in the heterozygous state (International PPH Consortium *et al.* 2000; Ragge *et al.* 2005). Therefore, not only miscarriage and birth defects, but also other human diseases can be induced by defective essential genes, including different types of cancer (Dickerson *et al.* 2011).

How many human orthologs of the essential genes in *C. elegans* that we identified are related to human disorders? Of the 143 essential genes that were gathered for the functional analysis, 108 were identified as having a putative ortholog in human. Among those 108 genes, 97 (90%) are associated with 1218 different diseases. For instance, *SF3B1*, which encodes an RNA-splicing factor and is orthologous to *let-763/sftb-1*, has mutations related to myeloid cancers and refractory anemias (Papaemmanuil *et al.* 2011; Broseus *et al.* 2013). In another case, the ortholog of F11A3.2 in human is a translation initiation factor EIF2B4, which is associated with the inheritable disease Vanishing White Matter (Kanbayashi *et al.* 2015). Moreover, 60 of the 97 genes have existing variations that were found to be responsible for 163 different diseases. Consistent with previous findings (Dickerson *et al.* 2011), mutations in these genes can lead to a broad spectrum of human disorders. The most abundant diseases tend to be related to cancer or carcinogenesis, including breast carcinoma and colorectal cancer, suggesting links between adult cellular abnormalities and developmental functions.

A large number of essential genes were molecularly identified in this study, which significantly enlarges the database of essential genes in *C. elegans*. The identified lethal alleles provide a rich resource for future studies on essential genes, considering the difficulty of isolating and maintaining lethal mutations. Moreover, conserved essential genes could be good gene models for developing our understanding of the functions of homologous genes in humans, especially disease-related genes. On the other hand, according to a recent study, the essential genes that are only conserved in nematodes can play critical roles in core eukaryotic processes, especially in chromosome segregation (Verster *et al.* 2017). Meanwhile, species-specific essential genes could be good candidates for targeting pathogenic nematodes. Considering the functional importance of essential genes, studies can be conducted on the ones that currently have no annotated functions.

## Supplementary Material

Supplemental material is available online at www.g3journal.org/lookup/suppl/doi:10.1534/g3.117.300338/-/DC1.
