# Supplementary material for: Genomic Identification and Functional Characterization of Essential Genes in Caenorhabditis elegans
Source: G3 (Bethesda). 2018 Jan 15;8(3):981–97. doi: 10.1534/g3.117.300338 (PMC5844317; doi:10.1534/g3.117.300338)
Supplement: Supplementary file 1 [file 981FileS1.pdf]

Table 1. List of 60 essential genes from 104 lethal mutations identified on chromosome III(mid) and chromosome V(left)

Allele	Chr	Variation	Position	Mutation	Amino acid effect	Amino acid change	Affected gene	Evidence	Evidence supporting lethal phenotype*	Comment
let-702(s2483)	III	SNV	7359885	G->A	nonsense	W>*	*hmgr-1(F08F8.2)*	PCR	*tm4368* and RNAi	*tm4368* curated and published.
let-702(s2813)	III	SNV	7360261	C->T	missense	P>S	*hmgr-1(F08F8.2)*	WGS		
let-712(s2439)	III	SNV	8169268	G->A	nonsense	W>*	*prp-8(C50C3.6)*	WGS	*rr40* and RNAi	*rr40* curated and published.
let-712(s2598)	III	SNV	8166973	G->A	nonsense	W>*	*prp-8(C50C3.6)*	WGS		
let-786(s2631)	III	SNV	8353810	C->T	missense	T>I	*unc-116(R05D3.7)*	WGS	*rh24* and RNAi	*unc-116* published.
let-727(s2589)	III	SNV	5482194	G->A	nonsense	W>*	*R02F2.7*	WGS	RNAi	
let-728(s2573)	III	SNV	6205725	G->A	nonsense	Y>*	*C23G10.8*	WGS	*gk3389* and RNAi	*gk3389* curated NOT published.
let-728(s2573)	III	SNV	6205892	T->A	missense	D>N	*C23G10.8*	WGS		
let-732(s2611)	III	SNV	4943615	C->T	missense	S>L	*rpb-2(C26E6.4)*	WGS	*ok2893* and RNAi	*ok2893* curated NOT published.
let-732(s2808)	III	SNV	4943991	C->T	nonsense	Q>*	*rpb-2(C26E6.4)*	WGS		
let-832(s2817)	III	SNV	5556761	G->A	missense	E>K	*hach-1(F09F7.4)*	WGS	RNAi	
let-736(s2577)	III	SNV	4337800	G->A	missense	R>Q	*cdk-12(B0285.1)*	WGS	*tm3846* and RNAi	*tm3846* curated NOT published.
let-741(s2810)	III	SNV	4914874	G->A	missense	E>K	*rars-1(F26F4.10)*	WGS	*gc47* and RNAi	*gc47* curated and published.
let-741(s2476)	III	SNV	4914975	G->A	missense	R>K	*rars-1(F26F4.10)*	WGS		
let-743(s2581)	III	SNV	7764786	T->A	nonsense	L>*	*ZK686.2*	WGS	*tm5978* and RNAi	*tm5978* curated NOT published.
let-743(S2830)	III	SNV	7765501	C->T	nonsense	Q>*	*ZK686.2*	WGS		
let-747(s2807)	III	SNV	6461537	G->A	nonsense	W>*	*algn-1(T26A5.4)*	PCR	RNAi	
let-747(s2456)	III	SNV	6461808	G->A	missense	D>N	*algn-1(T26A5.4)*	WGS		

let-752(s2619)	III	1Del	7706605	delG	frameshift		*lin-13(C03B8.4)*	WGS	*n770* and RNAi	*n779* curated and published.
let-753(s2605)	III	SNV	5226777	G->A	splice signal		*C34E10.10*	PCR	None	
let-753(s2614)	III	SNV	5227554	G->T	missense	D>Y	*C34E10.10*	WGS		
let-757(s2460)	III	1Ins	7631830	G	frameshift		*fbn-1(ZK783.1)*	WGS	*tm290* and RNAi	failed to complement *let-821*
let-757(s2867)	III	SNV	7638442	G->A	missense	C>Y	*fbn-1(ZK783.1)*	WGS		*tm290* curated NOT published.
let-821(s2804)	III	SNV	7626858	G->A	missense	C>Y	*fbn-1(ZK783.1)*	WGS		
let-763(s2580)	III	SNV	4267912	G->A	nonsense	W>*	*sftb-1(T08A11.2)*	WGS	RNAi	
let-764(s2616)	III	1Del	6932404	4nt deletion	frameshift		*byn-1(F57B9.5)*	WGS	*tm4451* and RNAi	*tm4451* curated NOT published.
let-771(s2442)	III	SNV	7022627	G->A	missense	G>E	*rfl-1(F11H8.1.2)*	WGS	*or198* and RNAi	*or198* curated and published.
let-774(s2615)	III	SNV	6207832	C->T	nonsense	Q>*	*rps-3(C23G10.3.2)*	WGS	RNAi	rescue assay
let-782(s2591)	III	SNV	4705658	G->A	missense	C>Y	*tag-189(T04A8.12)*	WGS	*gk285*	*gk285* curated NOT published.
let-784(s2602)	III	SNV	5260831	G->A	missense	E>K	*gop-3(C34E10.1.2)*	WGS	*tm3269* and RNAi	*tm3269* curated NOT published.
let-798(s2850)	III	SNV	5725120	T->A	nonsense	C>*	*wrm-1(B0336.1)*	WGS	*ne1982* and RNAi	*ne1982* curated and published.
let-799(s2852)	III	SNV	5851950	G->A	missense	G>E	*ddx-23(F01F1.7)*	WGS	RNAi	
let-826(s2820)	III	SNV	4936540	G->A	nonsense	W->*	*mrps-18C(C26E6.6)*	PCR	RNAi	
let-826(S2811)	III	SNV	4936112	G->A	nonsense	W->*	*mrps-18C(C26E6.6)*	WGS		
let-827(S2827)	III	1Del+1Ins	4913180	delGinsA	frameshift		*cee-1(F26F4.1)*	WGS	RNAi	

let-829(S2826)	III	SNV	5229181	G->A	missense	G>R	*atp-2(C34E10.6)*	WGS	*ua2* and RNAi	*ua2* curated and published.
let-972(s2441)	III	1Ins	8446444	G	frameshift		*hsp-110(C30C11.4)*	PCR	*gk533* and RNAi	*gk553* NOT curated.
let-972(s2583)	III	SNV	8446436	G->A	missense	V->M	*hsp-110(C30C11.4)*	WGS		
let-326(s1404)	V	SNV	1263600	C->T	nonsense	W>*	*rab-1(C39F7.4)*	WGS	*tm2603* and RNAi	*tm2603* curated NOT published.
let-326(s238)	V	SNV	1263843	G->A	missense	S>F	*rab-1(C39F7.4)*	WGS		
let-332(s234)	V	SNV	7243996	G->A	nonsense	Q>*	*C05C8.7*	WGS	RNAi	
let-332(s369)	V	SNV	7245765	G->A	missense	T>I	*C05C8.7*	WGS		
let-335(s1439)	V	SNV	7856077	A->T	nonsense	K>*	*C37C3.2*	WGS	RNAi	
let-335(s1476)	V	SNV	7856451	G->A	missense	C>Y	*C37C3.2*	WGS		
let-342(s1029)	V	SNV	4632154	G->A	missense	D>N	*pmt-2(F54D11.1)*	WGS	ok2419 and RNAi	*ok2419* curated NOT published.
let-342(s1442)	V	SNV	4631873	C->T	missense	S>F	*pmt-2(F54D11.1)*	WGS		
let-343(s1025)	V	SNV	7179311	G->A	missense	G>D	*cpsf-2(F09G2.4)*	WGS	RNAi	
let-343(s1428)	V	SNV	7180252	C->T	nonsense	Q>*	*cpsf-2(F09G2.4)*	WGS		
let-346(s1575)	V	SNV	7208502	G->A	nonsense	R>*	*soap-1(C13F10.4)*	WGS	*tm5803* and RNAi	*tm5803* curated NOT published.
let-346(s1619)	V	SNV	7209026	G->A	nonsense	Q>*	*soap-1(C13F10.4)*	WGS	*tm5803* and RNAi	
let-348(s1436)	V	SNV	4441757	G->A	missense	S>F	*rft-1(Y47D7A.16)*	WGS	RNAi	
let-348(s1622)	V	SNV	4439195	C->T	nonsense	W>*	*rft-1(Y47D7A.16)*	PCR		
let-402(s127)	V	SNV	6435516	G->A	missense	G>E	*erfa-1(T05H4.6)*	WGS	RNAi	
let-402(s1526)	V	SNV	6434336	C->T	missense	S>L	*erfa-1(T05H4.6)*	WGS		
let-410(s1565)	V	SNV	6708760	C->T	splice signal		*dlst-1(W02F12.5)*	WGS	RNAi	
let-410(s815)	V	SNV	6707665	G->A	missense	S>F	*dlst-1(W02F12.5)*	WGS		

let-415(s1505)	V	SNV	4826120	G->A	missense	S>F	*hsp-6(C37H5.8)*	WGS	*tm515* and RNAi	*tm515* curated NOT published.
let-415(s1525)	V	SNV	4825181	A->T	missense	I>Y	*hsp-6(C37H5.8)*	WGS		
let-419(s1483)	V	SNV	5051195	C->T	nonsense	Q>*	*pqn-51(K11D12.2)*	WGS	RNAi	
let-419(s1539)	V	SNV	5051774	C->T	nonsense	Q>*	*pqn-51(K11D12.2)*	WGS		
let-424(s1587)	V	SNV	8033082	C->T	nonsense	Q>*	*vars-1(ZC513.4)*	WGS	RNAi	
let-424(s384)	V	SNV	8033150	G->A	nonsense	W>*	*vars-1(ZC513.4)*	WGS		
let-428(s1070)	V	SNV	4691491	C->T	nonsense	R>*	*K03B4.1*	WGS	RNAi	
let-428(s1490)	V	SNV	4691043	G->A	missense	G>D	*K03B4.1*	WGS		
let-463(s2168)	V	rearrange ment	breakpoint: 5916530..5917732				*C04E6.11*	WGS	*tm4214*	*tm4214* curated NOT published.
let-439(s1407)	V	SNV	5940676	G->A	nonsense	W>*	*M03F8.3*	WGS	RNAi	
let-439(s1503)	V	SNV	5940035	G->A	missense	M>I	*M03F8.3*	WGS		
let-443(s1417)	V	SNV	5940509	C->T	nonsense	Q>*	*M03F8.3*	WGS		failed to complement *let-439*
let-440(s1411)	V	SNV	5306632	G->A	splice signal		*ncx-2(C10G8.5)*	WGS	*tm4571* and RNAi	*tm4571* curated NOT published.
let-440(s1440)	V	SNV	5306878	G->A	missense	E>K	*ncx-2(C10G8.5)*	WGS		
let-442(s1416)	V	SNV	7232261	A->T	nonsense	K>*	*C05C8.2*	WGS	RNAi	
let-442(s1430)	V	SNV	7231630	G->A	nonsense	W>*	*C05C8.2*	WGS		
let-455(s1447)	V	SNV	4184746	C->T	nonsense	W>*	*Y45G5AM.9*	WGS	None	
let-455(s1511)	V	SNV	4184551	C->T	nonsense	W>*	*Y45G5AM.9*	WGS		
let-422(s1548)	V	SNV	5684545	C->T	missense	G>R	*hmgs-1(F25B4.6)*	WGS	RNAi	
let-422(s1563)	V	SNV	5684347	G->A	missense	S>F	*hmgs-1(F25B4.6)*	WGS		

let-411(s1453)	V	SNV	7806113	G->A	nonsense	W>*	*xpo-1(ZK742.1)*	WGS	RNAi	
let-411(s1595)	V	SNV	7806685	C->T	nonsense	Q>*	*xpo-1(ZK742.1)*	WGS		
let-334(s383)	V	SNV	8540346	G->A	missense	P>S	*slc-17.8(F25G6.7)*	WGS	None	
let-334(s908)	V	SNV	8541157	G->A	missense	A>V	*slc-17.8(F25G6.7)*	WGS		
let-409(s1480)	V	SNV	8546796	G->A	missense	G>E	*asns-1(F25G6.6)*	WGS	RNAi	
let-409(s823)	V	large deletion	8545461..8547038				*asns-1(F25G6.6)*	WGS		
let-409(s1507)	V	SNV	8548170	T->A	nonsense	K>*	*asns-1(F25G6.6)*	PCR		
let-331(s1608)	V	SNV	4715842	C->T	missense	G>E	*prx-6(F39G3.7)*	WGS	RNAi	
let-331(s427)	V	large deletion	4718927..4719368				*prx-6(F39G3.7)*	WGS		
let-327(s1458)	V	SNV	1986666	C->T	nonsense	R>*	*T08B1.1*	PCR	RNAi	
let-327(s1496)	V	SNV	1986588	G->A	nonsense	Q>*	*T08B1.1*	WGS		
let-417(s1679)	V	1Del+2Ins	5317072 G-TC	G->C	frameshift	Y>*	*ceh-34(C10G8.6)*	WGS	*tm3733* and RNAi	*tm3733* curated and published.
let-417(s204)	V	SNV	5317187	C->T	missense	R>Q	*ceh-34(C10G8.6)*	WGS		
let-350(s2126)	V	rearrange ment	breakpoint: 4828640..4834790				*C37H5.5*	WGS	RNAi	
let-350(s250)	V	SNV	4829444	G->A	splice signal		*C37H5.5*	WGS		
let-338(s503)	V	1Del	4200817		frameshift		*rpac-40(H43I07.2)*	WGS	*ok3654* and RNAi	*ok3654* curated NOT published.
let-338(s1020)	V	rearrange ment	breakpoints: 4202166..4202306 + AA				*rpac-40(H43I07.2)*	WGS		
let-420(s1046)	V	SNV	4821165	G->A	missense	P>L	*adss-1(C37H5.6)*	WGS	RNAi	
let-420(s1058)	V	SNV	4821048	G->A	missense	G>E	*adss-1(C37H5.6)*	PCR		

let-447(s1654)	V	rearrange ment	breakpoint: 41000				*egl-8(B0348.4)*	WGS	WGS	*egl-8* published.
let-447(s1457)	V						*egl-8(B0348.4)*	complemen-tation test		
let-459(s1615)	V	SNV	4346291	C->T	splice signal		*hpo-18(F32D1.2)*	WGS	*ok3436* and RNAi	*ok3436* curated NOT published.
let-459(s1432)							*hpo-18(F32D1.2)*	complemen-tation test		
let-470(s1581)	V	SNV	9516186	G->A	splice signal		*F11A3.2*	WGS	RNAi	
let-470(s1629)	V						*F11A3.2*	rescue assay		
let-408(s827)	V	SNV	8132990	G->A	nonsense	W>*	*snap-1(D1014.3)*	WGS	*tm2068* and RNAi	*tm2068* curated NOT published.
let-423(s1550)	V	SNV	7832305	C->T	nonsense	Q>*	*mig-6(C37C3.6)*	WGS	*ev788* and RNAi	*ev788* curated and published.
let-344(s1555)	V	big deletion	4540156..4563301				*sos-1(T28F12.3)*	WGS	WGS	*let-344* is *let-341*, with multiple lethal alleles published.

* "Evidence supporting lethal phenotype shows that alleles or RNAi evidence from WormBase support lethal phenotypes. However, no direct complementation test performed between these alleles and our sequenced alleles.

(1) 12 genes (2 with only tm alleles) highlighted in green were published with lethal alleles. Five of those genes *unc-116*, *lin-13*, *egl-8, sos-1* and *mig-6* were published prior to (Johnsen and Baillie 1991; Stewart *et al.* 1998) for *let-786*, *let-752*, and (Johnsen and Baillie (1991) for *let-447, let-344* and *let-423*. Therefore, *let-786* is *unc-116*, *let-752* is *lin-13*, *let-447* is *egl-8, let-344 is sos-1* and *let-423* is *mig-6*. The other seven were first published in Stewart *et al.* (1998) and Johnsen and Baillie (1991) and therefore the 'let' names in those publications takes precedent. (2) 18 genes (all of the alleles listed are gk, tm, or ok) in black have known lethal alleles which have not been curated and characterized and whose correct names were first published in Stewart *et al.* (1998) or Johnsen and Baillie (1991). (3) 31 genes highlighted in red have no alleles in WormBase. Their gene names are published in Stewart *et al.* (1998) or Johnsen and Baillie (1991).

Table 2. A total of 86 strains with *eT1(III;V)*

Strain	*let-x(allele)*	Block[1]	Mapping zone
BC9247	*let-350(s2126)*	Not noted	10
BC9296	*let-407(s2122)*	Not noted	20B
BC9246	*let-417(s1679)*	Not noted	10
BC9306	*let-447(s1654)*	MEL egg let	1B1
BC9254	*let-349(s502)*	Early	11B1
BC9316	*let-338(s503)*	Mid[2]	8A2
BC9235	*let-420(s723)*	Sterile	10
BC9286	*let-408(s827)*	Egg[3]	17
BC9295	*let-409(s823)*	Early	19
BC9262	*let-410(s815)*	Mid	14
BC9314	*let-426(s826)*	Mid	5
BC9312	*let-326(s1404)*	Early[4]	4A
BC9248	*let-327(s1496)*	Slow[5]	6A
BC9280	*let-335(s1439)*	Early	16
BC9278	*let-335(s1476)*	Early	16
BC9275	*let-339(s1444)*	Early	15
BC9271	*let-339(s1469)*	Early/Mid	15
BC9292	*let-340(s1508)*	Early	19
BC9242	*let-342(s1442)*	Early	9B
BC9277	*let-343(s1428)*	Early/Mid	15
BC9225	*let-344(s1555)*	Mid	9A
BC9232	*let-345(s1452)*	Mid	9B
BC9315	*let-346(s1575)*	Mid/Late	15
BC9276	*let-346(s1619)*	Mid	15
BC9230	*let-348(s1436)*	Early	9A
BC9238	*let-348(s1622)*	Early	9A
BC9267	*let-402(s1526)*	Early	12A

[1] Block describes the developmental blocking stage of the recovered lethal alleles.

[2] Mid: Mid larval lethal (worm's length 0.3-0.5mm)

[3] Egg: Embryogenesis

[4] Early: Early larval lethal (worm's length 0.2-0.3mm)

[5] Slow: Slow developing

BC9266	*let-403(s1482)*	Early	12B
BC9311	*let-407(s1631)*	Hatch[6]	20B
BC9321	*let-409(s1480)*	Hatch	19
BC9263	*let-410(s1565)*	Mid	14
BC9282	*let-411(s1453)*	Mid	16
BC9281	*let-411(s1595)*	Mid	16
BC9255	*let-415(s1505)*	Late	10
BC9272	*let-415(s1525)*	Early	10
BC9243	*let-419(s1483)*	Early/Mid	10
BC9234	*let-419(s1539)*	Early/Mid	10
BC9289	*let-422(s1548)*	Early	11B5
BC9258	*let-422(s1563)*	Early	11B5
BC9283	*let-423(s1550)*	Hatches	16
BC9301	*let-424(s1587)*	Sterile	17
BC9233	*let-426(s1527)*	Early	5
BC9251	*let-428(s1490)*	Sterile	10
BC9250	*let-439(s1407)*	Early	11B2
BC9259	*let-439(s1503)*	Early	11B2
BC9318	*let-440(s1411)*	Hatch	10
BC9253	*let-440(s1440)*	Hatches	10
BC9310	*let-442(s1416)*	Early/Mid	15
BC9269	*let-442(s1430)*	Early/Mid	15
BC9319	*let-443(s1417)*	Early	11A1
BC3048	*let-444(s1418)*	Early	12A
BC9256	*let-444(s1459)*	Mid	12A
BC9313	*let-447(s1457)*	Maternal[7]	1B1
BC9226	*let-455(s1447)*	Early	8A2
BC9227	*let-455(s1511)*	Early	8A2
BC9240	*let-459(s1432)*	Not noted	9A
BC9257	*let-459(s1615)*	Maternal	9A
BC9336	*let-463(s2168)*	Not noted	11B

[6] Hatch: Worm's length shorter than 0.2mm

[7] Maternal: Maternal lethal

BC9341	*let-470(s1581)*	Mid	20B
BC9299	*let-470(s1629)*	Slow	20B
BC9309	*let-331(s1608)*	Slow	10
BC9221	*let-326(s238)*	Mid	4A
BC9302	*let-327(s247)*	Slow	6A
BC9308	*let-331(s427)*	Slow	10
BC9270	*let-332(s234)*	Egg	15
BC9274	*let-332(s369)*	Egg	15
BC9293	*let-334(s383)*	Mid	19
BC9290	*let-334(s908)*	Early	19
BC9224	*let-338(s1020)*	Mid	8A2
BC9298	*let-340(s1022)*	Mid	19
BC9241	*let-342(s1029)*	Mid	9B
BC9268	*let-343(s1025)*	Egg	15
BC9222	*let-345(s578)*	Mid	9B
BC9245	*let-350(s250)*	Sterile	10
BC9294	*let-405(s388)*	Mid	16
BC9252	*let-420(s1046)*	Sterile	10
BC9297	*let-424(s384)*	Sterile	17
BC9307	*let-428(s1070)*	Sterile	10
BC9331	*let-466(s1063)*	Maternal	8A2
BC9249	*let-466(s990)*	Maternal	8A2
BC9260	*let-349(s217)*	Early	11B1
BC9264	*let-402(s127)*	Mid	12A
BC9265	*let-403(s120)*	Mid/Late	12B
BC9284	*let-405(s116)*	Early	16
BC9287	*let-414(s114)*	Mid	17
BC9261	*let-417(s204)*	Early	10

Table 3. A total of 49 strains with *sDp3*

Strain	*let-x(allele)*	Block	Mapping zone
BC4148	*mel-31(s2438)*	Maternal	11.12
BC4149	*let-712(s2439)*	Early	1A
BC4152	*let-771(s2442)*	Sterile	5
BC4156	*let-722(s2448)*	Early	1B
BC4165	*let-719(s2455)*	Sterile	11
BC4166	*let-747(s2456)*	Mid	7
BC4170	*let-757(s2460)*	Not noted	2
BC4173	*let-766(s2463)*	Late	1B
BC4186	*let-741(s2476)*	Early	11.12
BC4211	*let-700(s2571)*	Mid	13
BC4213	*let-728(s2573)*	Mid	7
BC4216	*let-780(s2576)*	Sterile	9
BC4217	*let-736(s2577)*	Mid	13
BC4220	*let-763(s2580)*	Early	13
BC4221	*let-743(s2581)*	Early	2
BC4223	*let-972(s2583)*	Early	1A
BC4229	*let-727(s2589)*	Early	11
BC4231	*let-782(s2591)*	Late	12
BC4237	*let-844(s2597)*	Early	1A
BC4238	*let-712(s2598)*	Early	1A
BC4241	*let-783(s2601)*	Embryo	5
BC4242	*let-784(s2602)*	Late	11
BC4251	*let-732(s2611)*	Early	12
BC4254	*let-753(s2614)*	Early	11.12
BC4255	*let-774(s2615)*	Early	7
BC4256	*let-764(s2616)*	Early	13
BC4259	*let-752(s2619)*	Mid	2
BC4261	*let-733(s2621)*	Mid	12
BC4271	*let-786(s2631)*	Early	1A
BC4822	*let-823(s2792)*	Early	12
BC4823	*let-824(s2793)*	Late	11
BC4829	*let-815(s2799)*	Early	7
BC4834	*let-821(s2804)*	Early	2
BC4838	*let-732(s2808)*	Maternal	12
BC4840	*let-741(s2810)*	Early	11.12
BC4841	*let-826(s2811)*	Sterile	11.12
BC4843	*let-702(s2813)*	Early	4

BC4845	*let-834(s2815)*	Sterile	1A
BC4847	*let-832(s2817)*	Late	11
BC4850	*let-826(s2820)*	Sterile	11.12
BC4854	*let-797(s2824)*	Early	9
BC4855	*let-793(s2825)*	Maternal	5
BC4856	*let-829(s2826)*	Early	11.12
BC4857	*let-827(s2827)*	Sterile	11.12
BC4860	*let-743(s2830)*	Early	2
BC4864	*let-837(s2834)*	Mid	11.12
BC4880	*let-798(s2850)*	Embryo	9
BC4882	*let-799(s2852)*	Early	9
BC5768	*let-757(s2867)*	Not noted	2

Table 4. Strains used in the complementation tests

Strain	*Gene*	*Allele*	*Genetic balancer**
BC4170	*let-757*	*s2460*	*sDp3*
BC5768	*let-757*	*s2867*	*sDp3*
BC4834	*let-821*	*s2804*	*sDp3*
BC4149	*let-712*	*s2439*	*sDp3*
BC4283	*let-712*	*s2598*	*sDp3*
BC4225	*let-786*	*s2585*	*sDp3*
BC4271	*let-786*	*s2631*	*sDp3*
BC9225	*let-344*	*s1555*	*eT1;let-500*
BC9239	*let-344*	*s376*	*eT1;let-500*
BC1925	*let-341/sos-1*	*s1031*	*eT1*
BC2233	*let-459*	*s1432*	*eT1*
VC2645	*hpo-18*	*ok3436*	*nT1, CGC*
RM2221	*egl-8*	*md1971*	*discernable phenotype, CGC*
BC9313	*let-447*	*s1457*	*eT1;let-500*
BC9306	*let-447*	*s1654*	*eT1;let-500*
BC9298	*let-340*	*s1022*	*eT1;let-500*
BC9292	*let-340*	*s1508*	*eT1;let-500*
FX11145	*sup-37*	*tm356*	*eT1, NBRP*
BC9247	*let-350*	*s2126*	*eT1;let-500*
BC3556	*let-415*	*s1505*	*eT1*

Note*: *sDp3* and *eT1* used as balancers for isolating the lethal mutations. Some strains were crossed to *let-500* (which is an early blocking lethal on an *eT1* chromosome) to reduce the contribution of homozygous *eT1* containing worms. The knockout alleles (*ok3436*, *tm356*) and the phenotypic allele (*md1971*) were ordered from the National BioResource Project (NBRP)::*C. elegans* and the Caenorhabditis Genetics Center (CGC), which is funded by the NIH Office of Research Infrastructure Programs (P40 OD010440).

References:

Johnsen, R. C., and D. L. Baillie, 1991 Genetic analysis of a major segment [LGV(left)] of the genome of Caenorhabditis elegans. Genetics 129**:** 735-752.

Stewart, H. I., N. J. O'Neil, D. L. Janke, N. W. Franz, H. M. Chamberlin *et al.*, 1998 Lethal mutations defining 112 complementation groups in a 4.5 Mb sequenced region of Caenorhabditis elegans chromosome III. Mol Gen Genet 260**:** 280-288.
